# Membraneless Compartmentalization of Nuclear Assembly Sites during Murine Cytomegalovirus Infection

**DOI:** 10.3390/v15030766

**Published:** 2023-03-16

**Authors:** Hana Mahmutefendić Lučin, Silvija Lukanović Jurić, Marina Marcelić, Igor Štimac, Ivona Viduka, Gordana Blagojević Zagorac, Berislav Lisnić, Zsolt Ruzsics, Pero Lučin

**Affiliations:** 1 Department of Physiology and Immunology, Faculty of Medicine, University of Rijeka, 51000 Rijeka, Croatia; 2 Department of Histology and Embryology, Center for Proteomics, Faculty of Medicine, University of Rijeka, 51000 Rijeka, Croatia; 3 Institute of Virology, Faculty of Medicine, University Medical Center Freiburg, University of Freiburg, 79104 Freiburg, Germany

**Keywords:** beta-herpesviruses, cytomegalovirus, murine cytomegalovirus, nuclear replication compartment, membranelles organelles, biomolecular condensates, liquid–liquid phase separation

## Abstract

Extensive reorganization of infected cells and the formation of large structures known as the nuclear replication compartment (RC) and cytoplasmic assembly compartment (AC) is a hallmark of beta-herpesvirus infection. These restructurings rely on extensive compartmentalization of the processes that make up the virus manufacturing chain. Compartmentalization of the nuclear processes during murine cytomegalovirus (MCMV) infection is not well described. In this study, we visualized five viral proteins (pIE1, pE1, pM25, pm48.2, and pM57) and replicated viral DNA to reveal the nuclear events during MCMV infection. As expected, these events can be matched with those described for other beta and alpha herpesviruses and contribute to the overall picture of herpesvirus assembly. Imaging showed that four viral proteins (pE1, pM25, pm48.2, and pM57) and replicated viral DNA condense in the nucleus into membraneless assemblies (MLAs) that undergo a maturation sequence to form the RC. One of these proteins (pM25), which is also expressed in a cytoplasmic form (pM25l), showed similar MLAs in the AC. Bioinformatics tools for predicting biomolecular condensates showed that four of the five proteins had a high propensity for liquid–liquid phase separation (LLPS), suggesting that LLPS may be a mechanism for compartmentalization within RC and AC. Examination of the physical properties of MLAs formed during the early phase of infection by 1,6-hexanediol treatment in vivo revealed liquid-like properties of pE1 MLAs and more solid-like properties of pM25 MLAs, indicating heterogeneity of mechanisms in the formation of virus-induced MLAs. Analysis of the five viral proteins and replicated viral DNA shows that the maturation sequence of RC and AC is not completed in many cells, suggesting that virus production and release is carried out by a rather limited number of cells. This study thus lays the groundwork for further investigation of the replication cycle of beta-herpesviruses, and the results should be incorporated into plans for high-throughput and single-cell analytic approaches.

## 1. Introduction

Herpesviruses are a large family of DNA viruses that replicate in the nucleus and eventually assemble in the cytoplasm before leaving the infected cell. Members of the family are divided into three subfamilies, and the beta-herpesvirus subfamily includes four members known to infect humans. Human herpesvirus (HHV) 5 (HHV-5 or human cytomegalovirus, HCMV), HHV-6A, HHV-6B, and HHV-7 infect almost the entire human population and cause asymptomatic infection in immunocompetent individuals and enter a lifelong latent state from which they can be reactivated with as yet undetermined frequency [1]. Persistence and reactivation of CMV and other beta-herpesviruses have been associated with many pathophysiological conditions, and infection or reactivation in immunocompromised individuals can cause life-threatening disorders [1,2]. In addition, CMV is one of the most important known infectious causes of perinatal mortality, birth defects, and congenital anomalies [3]. Therefore, a comprehensive understanding of beta-herpesvirus biology and pathophysiology is essential for the development of effective preventive and therapeutic approaches. One of the critical points in the life cycle of beta-herpesviruses is the assembly of infectious virions.

Beta-herpesviruses extensively remodel the nucleus and cytoplasm, including membrane-bound organelles, to establish a production chain for the manufacture and release of newly formed virions (reviewed in [4,5,6,7]). Viral DNA transcription and replication require a complex machinery consisting of virus-encoded proteins and endogenous host cell processes [6]. Viral proteins required for DNA packaging and capsid production should be present and concentrated in sufficient quantities near the areas of DNA replication [6]. New capsids are assembled in the nucleus, exported to the cytosol, and enter the final stage of the manufacturing process, which includes secondary envelopment and packaging into the transport vehicles required for virion release from infected cells [4,5]. All processes in the manufacturing chain require spatiotemporal organization into units that can locally concentrate the molecules needed for the reaction at an appropriate concentration, as well as independent stores that would sequester excess proteins and serve as buffers for active units. When membrane organelles are involved, a mechanical force may also be required to shape the cell structures. These processes prove to be not very efficient and lead to a variety of outcomes, such as the production of large amounts of incomplete capsids and viral DNA-free capsids [8], the envelopment and release of tegument material without capsids (known as dense bodies) [4], or even the envelopment of DNA-free capsids [4]. To increase efficiency, all these processes require compartmentalization, which is achieved by the reorganization of membrane organelles and the formation of condensates without membranes, such as membraneless assemblies (MLAs) in the nucleus. Research interest in understanding compartmentalization of the cell is increasing exponentially, and it is now known that there are many pathways and purposes for compartmentalization (reviewed in [9,10,11,12]). One avenue is the development of biomolecular condensates by liquid–liquid phase separation (LLPS).

Most of the knowledge about beta-herpesviruses has been gained in studies of HCMV, supplemented by advances in studies of alpha-herpesviruses and complemented by studies of murine CMV (MCMV) infection. MCMV has proven to be an irreplaceable way to study the pathogenesis of CMV infection in animal models [13]. In addition, the study of MCMV infected cells offers many advantages because the replication cycle is shorter, especially under conditions of genetic or pharmacological suppression of host cell factors. Perturbation of host cell processes is similar in HCMV- and MCMV-infected cells, as shown by the establishment of the cytoplasmic assembly compartment (AC) [4,14] and the exploitation of egress pathways [15]. Thus, the same can be expected for the processes in the nucleus. Available information suggests that these processes are highly conserved in herpesviruses and even more so in DNA viruses [6], and studies in MCMV-infected cells may contribute to the overall understanding of nuclear compartmentalization. However, few studies have examined the nuclear processes during MCMV infection (e.g., [16,17,18,19,20,21]). Accordingly, there is a lack of characterization of many MCMV proteins that may contribute to these processes. Therefore, researchers using MCMV as a model are forced to infer nuclear events from an understanding of these processes in HCMV- or HSV-infected cells. Although the processes involved in HCMV nuclear replication are now much better understood, including tools developed to study nuclear events in cell biology, many fundamental questions remain unanswered, including spatiotemporal events and the biogenesis of nuclear compartmentalization [6].

In this study, we analyzed nuclear events during the early (E) and late (L) phases of infection. We analyzed replicated viral DNA by EdU labeling and five MCMV-encoded proteins: the immediate-early-1 protein (pIE1), the early-1 proteins (pE1s), the ss-DNA binding protein (pM57), the major tegument protein pM25 expressed in a smaller nuclear (pM25s) and a larger cytoplasmic (pM25l) form, and the small capsid protein (SCP, pm48.2). E phase lasts about 16 h [22,23] and involves the formation of cytoplasmic AC [14,24,25,26] and pre-replication compartments (pre-RCs) in the nucleus [16,17]. By 6 h post-infection (hpi), the immediate early (IE) genes (represented by pIE1) and the first set of early (E) genes (represented by pE1) are abundantly expressed [22,27,28], and the basic configuration of the cytoplasmic AC is established [14]. At later stages of the E phase (6-16 hpi), the second set of E genes (represented by pM57 and pM25s) is also abundantly expressed [16,17,22], and the cytoplasmic AC matures [14,29,30]. E phase is terminated 15–16 hpi by viral DNA replication [22], which is required for initiation of the L phase, expression of L genes, and formation of RCs. At 24–48 hpi, many structural proteins are expressed (represented by pm48.2 and pM25l [16,17,20]), and RCs and AC mature to establish a productive chain of virion assembly and release from infected cells [14,17]. Thus, by monitoring these five MCMV proteins and replicated viral DNA, we can track nuclear events, while monitoring pM25l can track maturation of the cytoplasmic AC. All these proteins were analyzed for their propensity to LLPS using the recently introduced package of bioinformatics tools [31], and LLPS analysis was extended to all MCMV proteins with nuclear localization. The sequence of visualized morphological changes in the nucleus and the bioinformatic analyses suggest that biomolecular condensation may be an essential mechanism for the reorganization of the infected cell.

## 2. Materials and Methods

### 2.1. Cells

Balb/3T3 cells (American Type Culture Collection, clone A31, ATCC CCL-163) were used for experiments and primary murine embryonic fibroblasts (MEFs) from 17-day embryos of BALB/c mice were used for virus production. Balb/3T3 cells were grown in DMEM supplemented with 10% fetal bovine serum (FBS), 2 mM L-glutamine, 100 mg/mL streptomycin, and 100 U/mL penicillin (all reagents from Gibco/Invitrogen, Grand Island, NY, USA). MEFs were grown in minimal essential medium (MEM) containing 5% FBS and the same supplements as DMEM. Cells were grown in Petri dishes and used for infection when they were 80–90% confluent.

### 2.2. Viruses and Infection Conditions

Preparation of MCMV stocks, infection of cells with MCMV, and quantification of viral titers were performed according to standard procedures [32]. Since our study is based on the extensive use of monoclonal antibody reagents (mAb), we infected cells with Δm138-MCMV (ΔMC95.15), a recombinant virus with the deletion of the fcr1 (m138) gene [33] generated on wild-type MCMV (strain Smith, ATCC VR -194). The ΔMC95.15 virus was used to avoid nonspecific capture of antibody reagents on infected cells by the m138 gene product, which has Fc receptor properties and is expressed early in infection. This virus has growth characteristics that are not different from wild-type virus [33], including the development of the assembly compartment, as described in our previous studies [14,24,25,26,30]. To visualize SCP, we used recombinant S-mCherry-SCP-MCMV, constructed on the MCMV-Δm1-16-FRT background, expressing mCherry-tagged SCP fusion protein [20]. Cells were infected with an infection multiplication (MOI) of 10, with an enhancement of infectivity by centrifugation [32], and infection efficiency was monitored by immunofluorescence detection of pIE1 as previously described [26].

### 2.3. Antibodies and Reagents

Monoclonal antibodies (mAbs) to MCMV proteins were produced, purified, and verified by the University of Rijeka Center for Proteomics (https://products.capri.com.hr/shop/?swoof=1&pa_reactivity=murine-cytomegalovirus (accessed on 11 November 2022)). For pIE1 (pm123), IgG_1_ (clone CROMA101; Cat. No. HR-MCMV-08) [34] and IgG_2a_ (clone IE1.01; Cat. No. HR-MCMV-12) [35] mouse mAbs were used; for E1 proteins (pM112-113), we used mouse IgG_1_ mAb (clone CROMA103; Cat. No. HR-MCMV-07) [34]; for pM25, we used mouse IgG_1_ mAb (clone M25C.01; Cat. No. HR-MCMV-03) [16]; for pM57, we used mouse IgG_1_ mAb (clone M57.02; Cat. No. HR-MCMV-6) [36]; and for pm06, we used mouse IgG_1_ mAb (clone CROMA229; Cat. No. HR-MCMV-02) [37].

Rabbit monoclonal IgG against Rab10 (Cat.No. 8127; Cell Signaling Inc, Danvers, MA, USA) was used in immunofluorescence, and mouse mAb against actin (Millipore, Burlington, Massachusetts, USA, Cat.No. MAB150) was used in Western blots. Alexa Fluor (AF)^488^-, AF^594^-, and AF^555^-conjugated secondary antibodies to mouse IgG_2a_, mouse IgG_1_, and rabbit IgG were from Molecular Probes (Leiden, The Netherlands), and AF^680^-conjugated anti-IgG_1_ and anti-IgG_2a_ were from Jacksons Laboratory (Bar Harbor, ME, USA).

1,6-Hexanediol (Cat. No, 240117-50G) was purchased from Sigma-Aldrich (St. Louis, Missouri, USA), heated to 45 °C, and dissolved to 50% stock solution in water. Other chemicals were from Sigma-Aldrich Chemie GmbH (Steinheim, Germany).

### 2.4. Immunofluorescence and Microscopy

Cells grown on coverslips were fixed with 4% formaldehyde (20 min at r.t.), permeabilized with 0.5–1% Tween 20 (20 min at 37 °C), incubated with primary antibodies (at least 60 min at r.t.), washed (three times 5 min at r.t.), and incubated with appropriate fluorochrome-conjugated secondary reagents (at least 60 min at r.t.). All antibody incubation and washing steps were performed in PBS containing 0.5% Tween 20. After washing three times in PBS, cells were embedded in Mowiol (Fluka Chemicals, Selzee, Germany)-DABCO (Sigma Chemical Co, Steinheim, Germany) in PBS containing 50% glycerol and analyzed by epifluorescence and confocal microscopy.

Samples were analyzed using an Olympus BX52 microscope (DP72CCD camera, cellSens Standard 1.15 software, magnification 400X).

Imaging was performed with either a Leica DMI8 inverted confocal microscope (confocal part: TCS SP8; Leica Microsystems GmbH, Wetzlar, Germany), equipped with a UV laser (Diode 405), Ar 488, DPSS 561, and He/Ne 633 lasers, or an Olympus Fluoview FV300 confocal microscope (Olympus Optical Co., Tokyo, Japan) equipped with Ar 488, He/Ne 543, and He/Ne 633 lasers. Images were acquired using LAS (Leica Application Suite) X Version 3.5.6.21594 software (Leica Microsystems GmbH, Wetzlar, Germany), HC PLAPO CS2 objective (63×1.40 oil), and 4 detectors (2xPMT and 2x HyD). Otherwise, images were acquired using Fluoview software, version 4.3 FV 300 (Olympus Optical Co., Tokyo, Japan), a PLAPO60xO objective, appropriate filters, and PMT detectors. The confocal aperture was set to 2. The z-series of 0.5 μm optical sections were acquired sequentially with an offset of less than 5% and medium scan speed (1.65 s/scan). Images (512 × 512 pixels) were acquired at different zoom values (zoom factor: 0.75−6.0) with pixel sizes ranging from 481.47 nm × 481.47 nm to 60.18 nm × 60.18 nm.

To avoid crosstalk between emission spectra, we used barrier filters for the Olympus FV300 microscope. For dual fluorescence, the interval was 510–530 nm for green fluorescence (AF488) and above 565 nm (AF594). For triple fluorescence, the interval was 510–530 nm for green fluorescence (AF488), 585–640 nm for red fluorescence (AF594), and above 660 nm for far-red fluorescence (AF680). For the Leica DMI8 microscope, the detectors were set to intervals of 500–535 nm for green fluorescence, 600–630 nm for red fluorescence, and 660–695 nm for far-red fluorescence.

### 2.5. Semiquantitative Analysis of Viral DNA Synthesis

Semiquantitative analysis of viral DNA synthesis was based on the incorporation of 5-ethynyl-2′-deoxyuridine (EdU) and its detection by a fluorescent azide using a Cu(I)-catalyzed [3 + 2] cycloaddition reaction [38]. The development of the assay was based on previous observations that MCMV infection inhibits cellular DNA synthesis in the E phase of infection [23]. To accurately test this observation, we infected 4 × 10^4^ cells/well in 24-well plates with Δm138-MCMV (MOI 10) and incubated them with 10 μM EdU at two-hour intervals from 2 to 24 h post infection. After 2 h of incubation, cells were fixed (4% PFA for 15 min at r.t.), washed three times with PBS containing 3% BSA (3% BSA-PBS), permeabilized for 20 min in 0.5% Triton X-100, washed three times with 3% BSA-PBS, and incubated for 30 min in the dark with 150 μL of the click-reaction cocktail using the Edu Click 555 ROTI kit for imaging (Carl Roth, Karlsruhe, Germany). After the reaction, cells were washed with 3% BSA-PBS and incubated with DAPI for 5 min. Cells were analyzed by confocal imaging of 10–15 randomly selected fields on the Olympus Fluoview FV300 confocal microscope (Olympus Optical Co., Tokyo, Japan) with 60x PlanApo objectives and 2x zoom (Z-axis 0.5 μm). The percentage of EdU-labeled cells was determined by quantifying the DAPI- and EdU-positive cells using an Olympus BX52 fluorescence microscope (DP72CCD camera, CellF software, magnification 400X).

### 2.6. Immunofluorescence and “Click Chemistry”

For simultaneous visualization of viral proteins, cells were incubated 16–24 hpi with EdU, fixed, permeabilized with 0.5% Triton X-100 and subjected to the Click reaction. After the Click reaction, cells were washed three times with PBS and incubated with 5 μg/mL of either anti-pE1, anti-pM57, or anti-pM25 in PBS containing 1% Tween for 60 min at r.t., followed by staining with AlexaFluor-488 (AF488)-conjugated anti-mouse IgG1.

### 2.7. Image Analysis

Images were exported as TIFF and analyzed using ImageJ 1.53c software without additional image processing. Focus plane images were used for image presentation.

Fluorescence intensity was quantified on focal plane images (pixel size 120.57 × 120.57 nm) using the Otsu Auto Threshold and Measure plugins [39]. Cells of interest were selected using the freeform selection tool, and the area, integrated density, and mean gray value were measured for each selected cell. The area adjacent to the selected cells that had no fluorescence was used for background correction. Total corrected cell fluorescence (TCCF) for each cell of interest was calculated using the following formula: integrated density − (area of selected cell × mean fluorescence of background values).

Three-dimensional (3D) analysis of colocalization was performed on images with pixel size of 120.57 × 120.57 nm using the JACoP plugin (http://rsb.info.nih.gov/ij/plugins/track/jacop.html, accessed on 7 September 2022) [40] to calculate the Manders′ overlap coefficients (M1 and M2) within the entire z-stack, as previously described [14].

The volume viewer plugin was used to reconstruct the 3D images of the entire z-series. The image sequence was imported into ImageJ, and the image stack is projected using the Volume mode and Trilinear interpolation. Z-Aspect and the offset were visually adjusted to eliminate the background and distinguish the spatial distribution.

### 2.8. Western Blot

Cell extracts for Western blot (WB) analysis were prepared in RIPA lysis buffer supplemented with protease and phosphatase inhibitors, separated by SDS-PAGE, and blotted at 100 to 130 V for 1 h onto a polyvinylidene difluoride (PVDF-P) WB membrane (Millipore, Burlington, Massachusetts, USA). Membranes were incubated with 1% blocking reagent (Roche Diagnostics GmbH, Mannheim, Germany) for 1 h, followed by a 1 h to overnight incubation with primary Abs, three wash cycles (TBS with 0.05% Tween 20 (TBS-T buffer)) and a 60-min incubation with peroxidase-conjugated secondary reagent diluted in TBS buffer containing 0.5% blocking reagent. After washing three times with TBS-T buffer (pH 7.5), the membranes were incubated with SignalFire Elite ECL reagent (Cell Signaling Technology, USA) for 1 min and enveloped in plastic wrap. Signals were detected using the Transilluminator Alliance 4.7 (Uvitec Ltd., Cambridge, UK).

WB signals were analyzed with Image J 1.53 software and normalized to the actin signal used as loading control. First, we calculated the normalization factor for each lane according to the formula: Lane Normalization Factor = Observed Actin Signal for each lane/Highest Observed Actin Signal for the blot. Thereafter, Normalized experimental signals were calculated as the Observed experimental signal/Lane normalization factor ratio.

### 2.9. Detection of Liquid–Liquid Phase Separation (LLPS) Drivers in MCMV Protein Sequences

Methods that identify disordered regions, domains, low-complexity regions (LCRs), short linear motifs (SLiMs), and stickers are complementary in detecting different LLPS drivers in protein sequences [31]. All protein sequences were retrieved from the UniProt database (Consortium UP, 2019; PMCID: PMC6323992) on 2 January 2022. Disorder region predictions of each protein were calculated using the VSL2 algorithm of the Predictor of Natural Disordered Regions (PONDR) platform (Molecular Kinetics, La Pas Trail, Indianapolis, IN, USA) [41], www.pondr.com (accessed on 2 January 2022). Low-complexity regions were predicted using SEG [42], available at https://mendel.imp.ac.at/METHODS/seg.server.html (accessed on 7 September 2022), with parameters 45/3.4/3.75. SLiMs were identified using the verified instances on the ELM server (ELM—the eukaryotic linear motif resource in 2020. (PMID:31680160), released 12 March 2020).

### 2.10. Prediction of LLPS Propensity in MCMV-Encoded Proteins

Five LLPS-specific prediction methods were used, as proposed by Pancsa et al., 2021 [31]. All were run on publicly available web servers with default settings after the sequence was uploaded in FASTA format.

PLAAC (Prion-Like Amino Acid Composition) detects disordered and prion-like domains (PLDs) with low sequence complexity using a Hidden Markov Model (HMM) algorithm [43]. It defines the region responsible for LLPS and does not specify a protein-level score. It was accessed at http://plaac.wi.mit.edu/ (accessed on 7 September 2022) and is considered a positive prediction if it yields a non-zero length region.

PSPer (Phase Separating Protein) allows prediction of whether a protein is likely to phase separate through RNA interactions (FUS-like proteins) and identification of regions within a protein that are mechanistically involved [44]. Accessed at https://www.bio2byte.be/b2btools/psp/ (accessed on 7 September 2022), and the cutoff value for the entire protein was 0.38, regardless of the region involved [31].

PScore is a machine learning-powered bioinformatic tool that predicts phase separation of intrinsically disordered protein regions (IDRs) based on the propensity for pi-pi contacts [45]. Accessed via http://abragam.med.utoronto.ca/~JFKlab/Software/psp.htm (accessed on 7 September 2022). PScore was considered positive if the value at any position in the sequence was greater than or equal to 4 [31].

catGRANULE is an algorithm for the prediction of LLPS propensity considering disorder propensity, RNA binding propensity and repetitive elements such as the content of RG -repeats (arginine-glycine) or FG -repeats (phenylalanine-glycine) [46]. Accessed via http://s.tartaglialab.com/new_submission/catGRANULES (accessed on 7 September 2022), evaluated by catGRANULE scores >0, indicating that a protein is prone to phase separation, and > 1, identifying high probability proteins prone to LLPS [47].

PSPredictor is a machine learning-based prediction model for phase-separating proteins (PSP) [48]. PSPredictor only assigns an overall score, but not regions. Accessed on http://www.pkumdl.cn:8000/PSPredictor/ (accessed on 7 September 2022).

### 2.11. Data Presentation and Statistics

Data are presented either as mean ± standard error of the mean (SEM) or as boxplots. Data comparison was performed using a two-tailed Student’s *t*-test when two samples were compared and one-way ANOVA analysis of variance for data with more than two experimental groups.

## 3. Results

### 3.1. Diffuse Nuclear Distribution of Immediate-Early 1 (IE1) Protein and Nuclear Membraneless Assemblies of Early 1 (E1) Proteins

Immediate-early 1 (pIE1, pm123) and early 1 (pE1, pM112-113) proteins are expressed very early after infection [18,19,21,22,27,28,49,50,51,52]. pIE1 is the product of the m123 gene, which belongs to the IE-class of regulatory proteins [49], while E1 proteins are the product of the M112-113 genes, which can generate at least four products of 33, 36, 38, and 87 kDa by alternative splicing [19,27], like their HCMV homologs UL112-113 [50].

**Figure 1:**
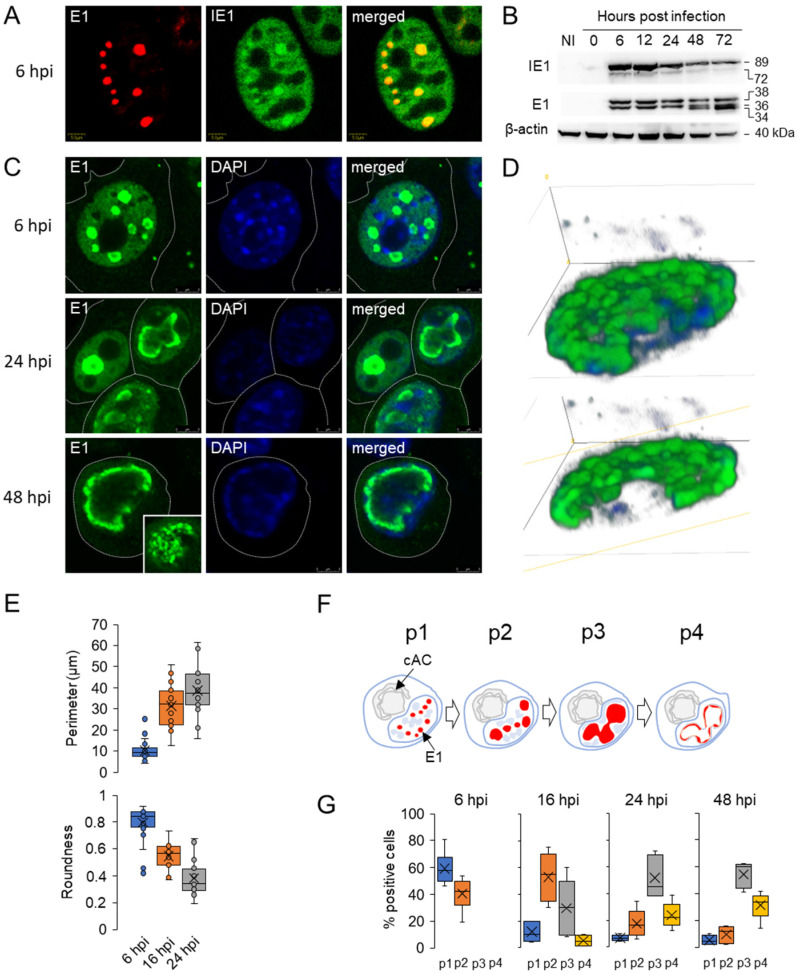
The maturation sequence of pE1 assemblies during MCMV infection. (**A**) Focal plane images of simultaneous visualization of pIE1 and E1 by antibody staining of Balb/3T3 cells at 6 hpi with Δm138-MCMV. (**B**) Representative WB analysis of the pIE1 and pE1s expression during MCMV infection. Quantitative analysis of expression level from five independent experiments is shown in Figure S2. (**C**) Focal plane images of representative pE1 patterns at 6, 24, and 48 hpi. pE1 proteins are visualized by antibody staining and DNA by DAPI. Inset, section through the peripheral plane. (**D**) The 3D reconstruction of pE1 assemblies at 48 hpi shown in the focal plane images in (**C**). (**E**) Average perimeter and roundness (4*area/(π*major_axis^2)) of E1 positive structures at 6 (*n* = 24 cells), 16 (*n* = 16), and 24 (*n* = 16) hpi determined by analysis of the focal plane images using Image J. (**F**) Schematic representation of the four identified patterns and maturation sequence of pE1 assemblies in the nucleus of MCMV-infected cells. (**G**) Percentage of MCMV-infected cells (identified by staining for pIE1) expressing the pE1 patterns determined after 6, 16, 24, and 48 hpi (11, 5, 5, and 7 experiments, respectively). At least 30 cells were analyzed in each experiment.

IE1 protein was detected as early as 2 h post infection (hpi) by immunofluorescence (Figure S1) and WB analysis (Figure S2). At 6 hpi (Figure 1A) and at later stages of infection (Figure S1), pIE1 was diffusely present in the nucleus and concentrated in distinct roundish areas of the nucleus. The pIE1 was intensively synthesized in the E phase of infection and its expression decreased at later stages of infection (i.e., 48 and 72 hpi, Figure 1B and Figure S2E).

M112-113 encoded transcripts are detected at 1 hpi [22] and pE1s can be visualized by immunofluorescence at 2–3 hpi [27]. Initial small spherical pE1s foci grew, formed nuclear clusters, and underwent a complex maturation sequence during the E and L phases of infection, similar to that described for their HCMV counterparts [53]. Representative images, schematic representations, and quantitative analyses of the maturation sequence are shown in Figure 1C–G. At the advanced stage of E phase (at 6 hpi), pE1s were identified in punctate nuclear patterns (Figure 1A,F-pattern p1) and in larger spherical assemblies in almost half of the cells (Figure 1C,E-p2), measured as high aspect ratio (roundness in Figure 1E). The number of these assemblies varied between 2 and 10 in mononucleated cells infected with MCMV at MOI of 10, and likely corresponds to the number of incoming genomes as described for herpes simplex virus 1 (HSV-1) and pseudorabies virus (PRV) infection [6,54,55]. At the end of E phase (16 hpi), the number in most cells decreased, and pE1 assemblies were much larger (Figure 1F-p3) with much higher perimeter and lower roundness (Figure 1E), indicating that they were fusing and growing. By 16 hpi, coincident with the onset of viral DNA synthesis [21,22], one-third of cells had pE1s condensed into one to three compact large MLAs that appeared to coalesce (Figure 1F-p3), and about 5% of cells had pE1s concentrated at the periphery with a hollow central region (Figure 1F-p4). The peripheral concentration of pE1s was observed in larger assemblies as early as 6 hpi (Figure 1C), suggesting that this event is initiated in the early stages of their maturation. At 24 hpi, the time at which the process of nucleocapsid production is fully developed and release of the virion from the cell begins, large MLAs (Figure 1C,F-p3) were the predominant pattern, with nearly 25% of cells exhibiting a single large hollowed nuclear domain (Figure 1C,F-p4,G). Surprisingly, similar patterns were observed at 48 hpi, a time of intense virion production and release (Figure 1C,G), suggesting that not all cells undergo maturation of pE1 MLAs to the large hollowed nuclear domain that might be expected as the final stage of their maturation.

The pE1s assemblies in the E phase of infection appear to be spherical structures, as confirmed by 3D reconstruction of the images. However, 3D analysis and reconstruction of the large hollow nuclear domain of pE1s, as seen in the fully developed domain in the 48-h infected cell in Figure 1C, revealed that pE1s was not homogeneously distributed at the periphery of the single nuclear domain but rather consisted of spherical MLAs (Figure 1D).

Overall, imaging analysis reveals the maturation sequence of pE1s MLAs during the replication cycle of MCMV. Consistent with previous studies of MCMV- [18,21] and HCMV- [53,54,55,56] infected cells, the small foci and spherical MLAs in the nucleus represent pre-replication centers (pre-RCs), while large globular domains and a single nuclear domain represent the replication compartment (RC). In contrast to pE1s, pIE1 remained scattered in the nucleus during 6–48 hpi, although pIE1 also concentrated within pE1-loaded spherical MLAs (Figure 1A). This is similar to the described behavior of IE1 and IE2 proteins in HCMV-infected cells [57] and IE3 protein in MCMV-infected cells [18,21,51].

### 3.2. Nuclear Membraneless Assemblies of pM57

**Figure 2:**
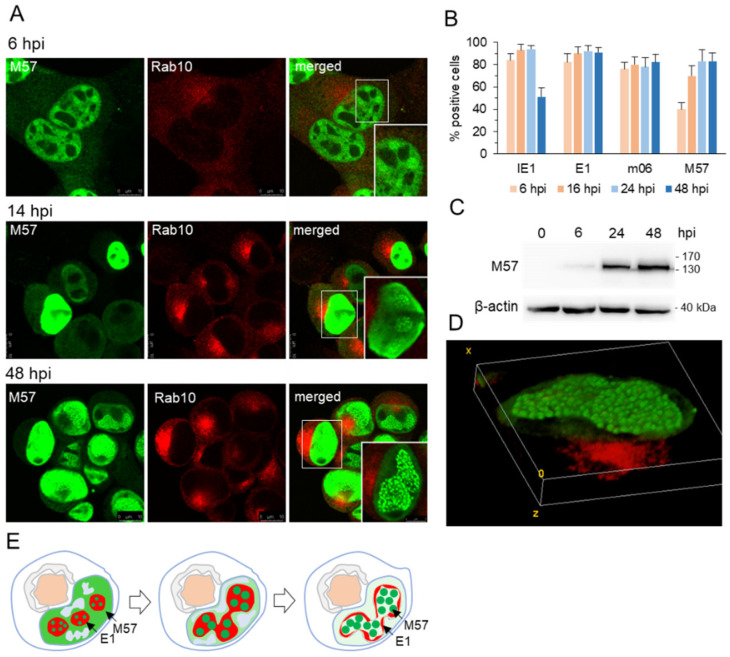
Analysis of pM57 expression and localization in MCMV-infected cells. (**A**) Balb/3T3 cells were infected with Δm138-MCMV at MOI of 10, fixed at different time points, and stained for immunofluorescence analysis with mouse IgG_1_ mAb against pM57 and rabbit mAb against Rab10, followed by staining with noncross-reactive fluorochrome-conjugated secondary Ab reagents and confocal microscopy analysis. Shown are representative images (focal plane through the middle portion of the cells). Insets show the boxed area imaged at higher magnification. Bars, 10 μm. (**B**) Percentage of DAPI-stained cells expressing IE1, E1, m06, and M57 as determined by immunofluorescence staining at different time points after infection. (**C**) Representative WB analysis of pM57 expression during MCMV infection. The quantitative blot analysis and average expression level from five independent experiments are shown in Figure S3. (**D**) 3D reconstruction of the whole Z-stack (17 layers of 0.5 µm each) with Image J Volume Viewer plugin of M57 (green) and Rab10 (red) stained cells shown in the inset of the 48 hpi-infected cell. (**E**) Schematic representation of the sequence of nuclear pM57 expression during the MCMV replication cycle.

MCMV pM57, homologous to HCMV UL57 and HSV ICP8, is expressed in the E phase of infection with delayed early kinetics [22,28]. It can be detected by immunofluorescence as early as 4–5 hpi, and at 6 hpi, it was detected in the nucleus (Figure 2A) of ~40% of infected cells (Figure 2B). WB analysis confirmed a low level of pM57 protein at 6 hpi (Figure 2C), corresponding to approximately 10–15% of total protein expression in infected cells (Figure S3). In one-third of these cells, pM57 showed diffuse nuclear staining with distinct large pM57-negative areas and bright circular pM57-positive accumulations (Figure 2A). Based on the studies of pUL57 [55], these bright areas correspond to pM57 accumulation in spherical MLAs that contain pE1 (Figure 1) and represent pre-RCs. Nuclear expression of pM57 precedes the establishment of cytoplasmic pre-AC, as shown by the simultaneous visualization of juxtanuclear Rab10 accumulation (Figure 2A) [14].

As E phase progressed (to 16 hpi), pM57 continued to accumulate in the nucleus, consistent with the continuous transcriptional activity of the M57 gene [22] and the accumulation of pM57 in the cells (Figure 2C). At the end of E phase (16 hpi), 75% of cells expressed pM57 (Figure 2B) diffusely in the nucleus (Figure 2A), but condensation to MLAs began in the 2–3 restricted areas (Figure 2A at 14 hpi) corresponding to pre-RCs formed prior to viral DNA replication which is expected from 15–16 hpi [22].

As infection progressed, the percentage of infected cells expressing pM57 increased to 80–85% (Figure 2B), suggesting that a small fraction of IE1-expressing infected cells were unable to overcome the E phase of infection to express late-early genes. Similar was observed for pm06 (Figure 2B) which belongs to the second set of E genes. Increased expression of pM57 was observed by WB analysis at 24 hpi and 48 hpi (Figure S3), suggesting that expression of pM57 persists to later infection time points. After 24 hpi and 48 hpi (Figure 2A), most of the diffuse nuclear staining of pM57 had disappeared, and most pM57 condensed to bright spherical MLAs within the large nuclear domain, like pICP8 [58] and pUL57 [56] in RCs of HSV-1- and HCMV-infected cells, respectively. Three-dimensional reconstruction of the images showed the dense accumulation of pM57 spherical structures within RCs (Figure 2D). These data suggest that pM57 staining reveals the final stage of maturation of pre-RCs and fully formed RCs of MCMV-infected cells from 16 hpi. Staining of pM57 with Rab10 could be a useful marker set to simultaneously visualize nuclear RCs and cytoplasmic AC.

### 3.3. Nuclear and Cytoplasmic Membraneless Assemblies of pM25

**Figure 3:**
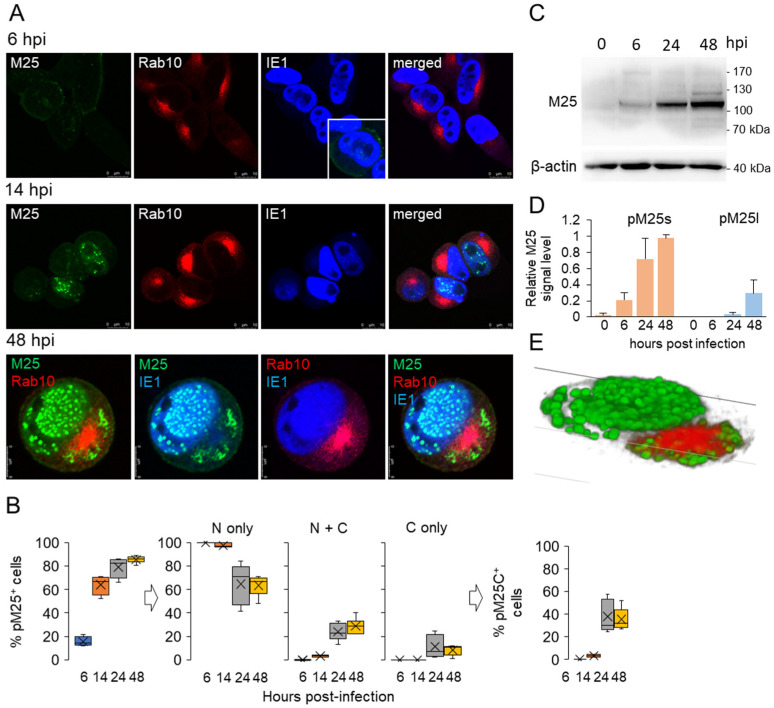
Analysis of pM25 expression and localization in MCMV-infected cells. (**A**) Balb/3T3 cells were infected with Δm138-MCMV at MOI of 10, fixed at different time points, and stained for immunofluorescence analysis with mouse IgG_1_ mAb against pM25, rabbit mAb against Rab10, and mouse IgG_2a_ mAb against IE1 (pm123), followed by staining with non-cross-reactive fluorochrome-conjugated secondary Abs and confocal microscopy. Shown are representative images through the focal plane. Cell boundaries are indicated by fine dotted lines. Bars, 10 μm. (**B**) Percentage of IE1-positive cells expressing pM25 in the nucleus (M25N), cytoplasm (M25C), or both (M25N+C) at 6, 14, 24, and 48 hpi. (**C**) Representative WB analysis of pM25 expression during MCMV infection. Quantitative analysis of the blot is shown in Figure S4. (**D**) The average expression level of 105 kDa pM25 (pM25s) and 130 kDa pM25 (pM25l) determined by analysis of four independent WB experiments. Data represent mean ± SEM values. (**E**) 3D reconstruction of the whole Z-stack (16 layers of 0.5 µm slices) using Image J Volume Viewer plugin of M25 (green) and Rab10 (red) stained cells shown in image (**A**) of 48 hpi-infected cells.

In contrast to M57, the expression kinetics and localization of M25 have been analyzed in more detail in previous studies [16,17]. MCMV M25 encodes a non-structural protein (105 kDa pM25s) expressed in the E phase and an abundant tegument protein (130 kDa pM25l) expressed in the L phase of infection [16,17,59,60]. The pM25 was identified at 6 hpi in the discrete nuclear condensates (Figure 3A) of less than 20% of infected cells (Figure 3B, left panel), without M25-specific staining in the perinuclear region. This is consistent with the WB analysis, which detected 105 kDa pM25 at 6 hpi (Figure 3C) [16], accounting for 20% of the total pM25 signal in infected cells (Figure 3D). Simultaneous staining with Rab10 showed that pM25-positive cells exhibited perinuclear Rab10 accumulation (Figure 3A, 6 hpi), which is characteristic of early rearrangement of membranous organelles that form the inner region of the cytoplasmic pre-AC [14,26]. However, many cells with Rab10 rearrangement were negative for pM25, suggesting that the cytopathogenic effects of pM25 expression associated with cell rounding and cytoskeletal reorganization [16] are not required for endosome rearrangement and that the MCMV-encoded function required for the establishment of the pre-AC is expressed before the M25 gene.

As infection progressed, pM25 continued to accumulate in the nucleus and developed larger MLAs, as shown by staining at 14 hpi (end of E phase) and 48 hpi (Figure 3A). At 48 hpi, pM25 was found in the cytosol of some cells in the large area surrounding the perinuclear accumulation of Rab10 (Figure 3A), suggesting that it accumulates in the outer region of AC, which is loaded with the Golgi membranes and viral glycoproteins [14]. Three-dimensional reconstruction of the example cell (48 hpi) shown in Figure 3A revealed large spherical accumulations of pM25 in both the nucleus and outer AC (Figure 3E).

At advanced stages of infection, ~80–85% of infected cells expressed pM25 (80.29 ± 8.23% at 24 hpi and 85.57 ± 2.86% at 48 hpi) (Figure 3B, left panel), resulting in an increase in pM25 detection by WB analysis (Figure 3C,D). At 48 hpi, the 130 kDa pM25 was detected in all WBs, accounting for approximately 25% of the total amount of pM25 in the cells, whereas the remainder was 105 kDa pM25. In the L phase, most pM25-positive cells expressed pM25 only in the nucleus (64.68 ± 17.58% at 24 hpi and 63.47 ± 8.67% at 48 hpi), a significant proportion of cells in both the nucleus and cytoplasm (24.05 ± 7.58% at 24 hpi and 28.65 ± 6.74% at 48 hpi), and a small proportion in the cytoplasm only (11.24 ± 10.05% at 24 hpi and 7.58 ± 5.43% at 48 hpi) (Figure 3B, middle panel). Since pM25 is the dominant protein in the virion tegument [59] and is required for virion infectivity [16], the cytoplasmic fraction of pM25 is essential for the final phase of cytoplasmic envelopment of MCMV. In our previous study of the AC, we showed that cytoplasmic MLAs of M25 appear as wrapped by membranes loaded with viral glycoproteins in the outer AC, suggesting a contribution of M25 to the final MCMV envelopment [14]. Therefore, from the expression patterns of pM25, we can conclude that no more than 30–40% of M25-positive cells (40.11 ± 15.35% at 24 hpi and 35.23 ± 9.6% at 48 hpi; Figure 3B, right panel) can contribute to the final stage of MCMV envelopment and production of fully mature MCMV virions.

Spatiotemporal analysis of the products of the M25 gene (Figure 3) reveals both nuclear and cytoplasmic events in host cell restructuring during MCMV infection. The cytoplasmic pattern is similar to that observed for HCMV UL25. However, UL25 does not localize in the nucleus, suggesting that the accumulation of pM25 in the nucleus is specific for MCMV infection and may play a role in nuclear RCs, similar to the function of the M25 homolog in HSV-infected cells.

### 3.4. Nucleocapsid Assembly Sites and Nuclear Assemblies of the Small Capsid Protein

**Figure 4:**
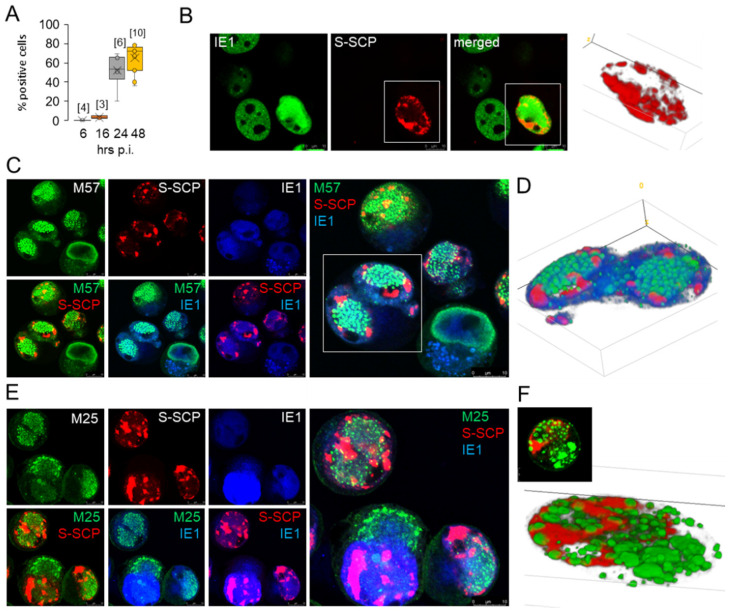
Analysis of SCP (pm48.2) expression and localization in MCMV-infected cells. (**A**) Percentage of IE1-positive cells expressing S-mCherry-SCP at 6, 16, 24, and 48 hpi. (**B**) Balb/3T3 cells were infected at MOI of 10 with S-mCherry-SCP-MCMV, fixed after 48 hpi, and stained for immunofluorescence analysis with mouse IgG_1_-mAb against IE1 (pm123), followed by staining with fluorochrome-conjugated secondary Ab and confocal microscopy analysis. Shown are representative images through the focal plane. The boxed area was zoomed, and the entire Z-stack (15 slices of 0.5 µM) was used for 3D reconstruction using the Volume Viewer plugin of ImageJ. (**C**) Representative images of S-mCherry- SCP-MCMV infected cells after 48 hpi stained with IgG_1_-mAb against pM57 and IgG_2a_ mAb against pIE1. Bars, 10 μm. (**D**) 3D reconstruction of the entire Z-stack (16 layers of 0.5 µM) of the zoomed boxed area shown in C using the Volume Viewer plugin of ImageJ. (**E**) Representative images of S-mCherry- SCP-MCMV infected cells at 48 hpi stained with IgG1 mAb against M25 and IgG_2a_ mAb against IE1. (**F**) The 3D reconstruction of the whole Z-stack (14 layers of 0.5 µM) of cells expressing S-mCherry-SCP and stained with anti-M25 using the Volume Viewer plugin of ImageJ.

To visualize the localization of nucleocapsid assembly sites, we infected cells with S-mCherry-SCP-MCMV, a recombinant virus expressing mCherry-tagged SCP encoded by m48.2 ORF, which is indistinguishable from wild-type MCMV in its infectivity and growth kinetics [20]. When infected with this virus at MOI of 10, a substantial amount of virus-associated pM45 was introduced into the cells (Figure S5), but no S-mCherry-SCP signal was detected in the cytoplasm. The percentage of IE1- and E1-expressing cells and their subcellular distribution were similar to those after Δm138-MCMV infection. A punctate mCherry fluorescence in the nucleus of a small number of pIE1-expressing cells was observed at 16 hpi and in ~50% at 24 hpi (Figure 4A). At 48 hpi, the S-mCherry-SCP immunofluorescence signal increased markedly compared with 24 hpi and appeared as large S-mCherry-SCP nuclear MLAs displaced to the periphery of the nucleus (Figure 4B). However, the percentage of pIE1-positive cells expressing S-mCherry-SCP did not change significantly at 48 hpi (Figure 4A), suggesting that not all infected cells complete all steps required for capsid component expression.

We further analyzed the subnuclear distribution of S-mCherry-SCP by simultaneous visualization with pIE1 and pE1 (not shown), pM57 (Figure 4C,D), and pM25 (Figure 4E,F). At 48 hpi, the S-mCherry-SCP signal (Figure 4B–F) was mainly confined to the periphery or outside the pM57- or pM25-positive RCs, and tiny punctate S-mCherry-SCP signals appeared to emanate from the patches toward the central area of the RCs. The 3D reconstruction of two cells in Figure 4D and 4F shows predominantly spherical MLAs of pM57 and pM25 and large non-spherical S-mCherry-SCP MLAs forming at the edges of the RCs but spreading mainly toward the periphery of the nucleus. This appearance is reminiscent of that of VP26 in HSV-1-infected cells [58], which initially forms small foci at the periphery of RCs that expand over time and coalesce into much larger foci that are pushed to the periphery of the nucleus as infection progresses. However, 3D reconstruction showed that the pM25-positive MLAs were separate entities embedded in a large mCherry-pm48.2-positive matrix (Figure 4E,F). In cells expressing pM25 in the cytoplasm, large spherical pM25 MLAs appeared to coalesce and fuse in the area of cytoplasmic AC, but no S-mCherry-SCP fluorescence was detected in these areas (Figure 4F).

Overall, our analysis demonstrates that large amounts of S-mCherry-SCP were synthesized in the infected cells and the bulk of the protein localized to the peripheral nuclear area outside the RCs. Of note, in cells with high cytoplasmic and low nuclear expression of pM25 together with S-mCherry-SCP MLAs, we frequently observed pIE1 staining in the juxtanuclear region of the cytoplasm, as shown in Figure 4E. This staining did not overlap with cytoplasmic pM25, indicating localization of pIE1 to the inner AC. The cytoplasmic pIE1 was likely observed due to binding of IgG_2a_ mAbs used for visualization of pIE1 by the pm138, which has FcR properties [33], as the S-mCherry-SCP-MCMV used for infection in these experiments was constructed on an MCMV-Δm1-16- FRT background, a virus with deleted first 16 genes but containing m138 [20].

### 3.5. Viral DNA Synthesis and Visualization of Viral DNA within Nuclear Replication Compartments

**Figure 5:**
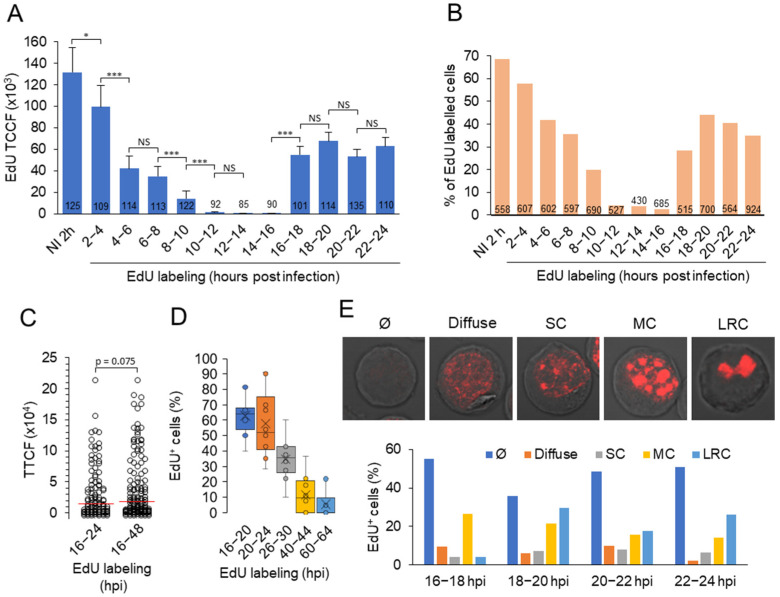
Analysis of DNA replication in MCMV-infected cells. (**A**) Non-infected (NI) and Δm138-MCMV-infected Balb/3T3 cells (MOI of 10) were incubated with 10 µM EdU at 2-h intervals from 2 to 24 hpi, fixed, permeabilized, and stained with the EdU-Click-555 click reaction cocktail. Cells were analyzed by confocal imaging of 10–15 randomly selected fields, and focal plane images were quantitatively analyzed using Image J. Total corrected cell fluorescence (TCCF) was calculated for each cell. The number of cells analyzed is indicated in the bars. Data are mean±SEM; *** *p* ˂ 0.001, * *p* ˂ 0.05 (one-way ANOVA). (**B**) Percentage of EdU-labeled cells determined by quantification of DAPI- and EdU-positive cells in the experiment described in A under epifluorescence microscope. The number of cells analyzed is indicated within or above the bars. (**C**) EdU labeling at 16–24 and 16–48 hpi. The amount of incorporated EdU was quantified on confocal images in the focal plane (0.5 µm) as total corrected cell fluorescence (TCCF), and the average value of 90–125 cells on 10–15 fields was determined. (**D**) EdU incorporation at different time points of MCMV replication. The percentage of EdU-labeled cells was determined by quantifying the DAPI- and EdU-positive cells on 10 randomly selected fields from two experiments. (**E**) Pattern of EdU incorporation into nuclei of MCMV-infected cells labeled 16–20 hpi with EdU. Ø, no EdU incorporation; Diffuse, diffuse EdU incorporation; SC, small condensates; MC, multiple small condensates; LRC, large replication centers. The percentage of cells showing the five patterns of EdU incorporation is shown below.

Early studies using ^3^H-thymidine incorporation [23] and Southern blot analysis of concatemeric DNA [22] reported that synthesis of MCMV DNA begins 15–16 hpi in infected fibroblasts, but there is no study visualizing viral DNA in RCs. HCMV DNA replication has been visualized in several studies by labeling with bromodeoxyuridine (BrdU) [55] and 5-ethynyl-2-deoxyuridine (EdU) [56]. In this study, we labeled replicated DNA with EdU and visualized incorporated EdU by the click chemistry reaction with fluorescent azide [38], a procedure that does not alter cellular proteins and can be combined with antibody staining and visualization. To make use of this labeling for visualization of the replicated viral DNA, it was essential to confirm that MCMV infection completely blocks DNA replication in the host cell during the E phase of infection, as it has long been unclear whether cellular DNA replication is inhibited in CMV-infected cells [55]. Therefore, we labeled infected cells with EdU for two hours throughout the 2–24 hpi period (Figure 5A,B). After 2-h exposure, EdU was taken up by 69% of uninfected cells (Figure 5B). In MCMV-infected cells, the EdU fluorescence signal and the number of EdU-incorporating cells decreased as early as 2 to 4 hpi, followed by a marked decrease at 4 to 6 hpi that persisted until 10 to 12 hpi (Figure 5A,B). Almost no EdU incorporation was detected in the 10–12, 12–14, and 14–16 hpi periods, suggesting that MCMV infection disrupts host cell DNA synthesis in the E phase of infection. This disruption is gradual and occurs from 2–10 hpi. At 16–18 hpi, EdU incorporation increased and can be determined at two-hour intervals up to 24 hpi (Figure 5A,B), consistent with the known kinetics of MCMV DNA synthesis and L gene expression in MEF and NIH 3T3 cells [22,28]. Thus, EdU incorporation clearly distinguishes between the disruption of DNA synthesis in the host cell and the onset of viral DNA synthesis, as also found by the ^3^H-thymidine incorporation assay [23]. The majority of cells replicating viral DNA initiated replication 16–24 hpi, and almost no difference in EdU incorporation was detected when cells were labeled with EdU between 16–24 hpi and 16–48 hpi (Figure 5C). Viral DNA replication was detected in approximately 60% of infected cells labeled with EdU at 16–20 and 20–24 hpi (Figure 5D). At later times (26–30 hpi), the percentage of labeled cells decreased, and very little EdU incorporation was detected at 40–44 or 60–64 hpi (Figure 5D). These data, together with EdU labeling at 16–48 hpi (Figure 5C), indicate that most of the replication events are initiated at 16–20 hpi and that DNA replication continues beyond 30 hpi.

Imaging of infected cells revealed several patterns of EdU distribution in the nucleus, including pale diffuse nuclear incorporation, punctate small condensates arising from diffuse nuclear staining, multiple condensates that grow and coalesce, and one, two, or three large clusters of replicated viral DNA representing RCs (Figure 5E). These patterns were observed in the 16–18 hpi stained cells and at later time points (22–24 hpi) the presence of large clusters was the predominant pattern, present in approx. 25% of cells (Figure 5E). These patterns are similar to those described for cells infected with HSV-1 [58,61] and HCMV [55,56] and suggest that viral DNA is replicated in different areas of the nucleus. The replicated DNA-containing condensates grow, coalesce, and fuse over time to form the large RCs as described in HSV-infected cells [61].

**Figure 6:**
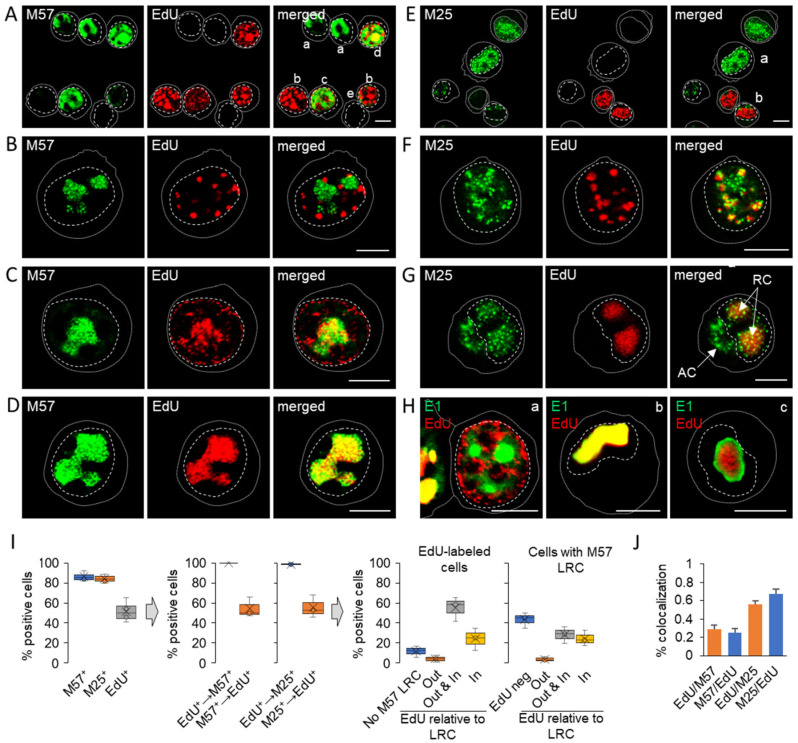
Simultaneous visualization of replicated viral DNA with pM57, pM25, or pE1. Cells were labeled 16–24 hpi with EdU and stained with antibodies against pM57 (**A**–**D**) or pM25 (**E**–**G**) or pE1 (**H**). Patterns of pM57 and EdU staining are shown in (**A**) (marked as a–e), patterns of pM25 and EdU staining are shown in (**E**) (marked as a–b), and representative higher magnification confocal images are shown below (**B**,**D** and **F**,**G**, respectively). The patterns of EdU and pE1 (marked as a–c) are shown in **H** as merged images. Cell borders are indicated by fine dotted lines and nuclei by fine dashed lines. Bars, 10 μm. AC, cytoplasmic assembly compartment; RC, nuclear replication compartment. (**I**) Percentage of pM57-, pM25-, and EdU-labeled cells and quantification of observed patterns were determined in at least three independent experiments. LRC, large replication compartment. (**J**) 3D colocalization of EdU with pM57 or pM25 based on M1/M2 coefficients of pixel overlap measured across the Costes-algorithm thresholded z-stacks of confocal images. Data represent mean±SEM per cell (n = 12–25).

To understand the relationship between viral DNA replication centers and RC, we performed a systematic analysis of the simultaneously visualized EdU-labeled nuclear condensates and RCs by staining three viral proteins (pM57, pM25, and pIE1) expressed in the E phase of infection (Figure 6A–H). Simultaneous visualization of EdU and pM57 at 24 hpi revealed cells with fully developed pre-RCs but without EdU labeling (Figure 6A-a), EdU labeling with low pM57 staining (Figure 6A-b), EdU labeling outside the pM57-positive pre-RCs (Figure 6A-c and 6B,C), EdU accumulation within fully developed M57-positive RCs (Figure 6A-d and 6C,D), and infected cells with cytopathogenic rounding without EdU labeling and pM57 staining (Figure 6A-e). A similar pattern was observed after visualization of pM25 nuclear MLAs (Figure 6E–G), including colocalization of EdU-labeled DNA within pM25-positive MLAs that did not organize into LRCs (Figure 6F) and within LRCs (Figure 6G), resulting in a high degree of colocalization of EdU and pM25 (Figure 6J). In cells with scattered pM25 MLAs, pM25 was rarely observed in the cytoplasm, whereas in cells that condensed EdU-labeled viral DNA and pM25 MLAs into large RCs (Figure 6G, RC), pM25 was frequently observed in cytoplasmic MLAs within AC (Figure 6G, AC). Simultaneous visualization of EdU-labeled DNA and pE1s also revealed viral DNA replication outside (Figure 6H-a) and in most cells within pE1-positive RCs (Figure 6H-b and c).

Approximately 80–85% of infected cells organized pM57- and pM25-positive MLAs (Figure 6I, left panel) in clusters representing large RCs (LRCs), similar to pE1 (Figure 1F, p3, and p4). However, only half of the pM57- and pM25-positive cells showed condensates of EdU-labeled viral DNA, and almost all EdU-positive cells exhibited pM57- and pM25-positive MLAs (Figure 6I, middle panel). However, EdU labeling did not depend on the LRCs formed. EdU incorporation and condensation were observed in ~12% of cells that did not develop pM57-positive LRCs (Figure 6I, right panel), in ~4% of cells outside pM57-positive LRCs (Figure 6B,I-right panel), and in ~55% of cells both outside and inside pM57-positive LRCs (Figure 6C,I-right panel). Only ~24% of cells showed EdU condensates within the LRCs at 24 hpi (Figure 6D,I-right panel). At these sites, pM57 and EdU-labeled viral DNA colocalized as expected (Figure 6C,D). However, both pM57- and EdU-labeled viral DNA formed globular MLAs that did not overlap, as shown in Figure 6C, suggesting a mechanism of their partitioning within RCs. In contrast, EdU-labeled viral DNA MLAs that evolved outside or in the absence of pM57-positive pre-RCs showed much larger spherical shapes, as shown in Figure 6A (shape b) and Figure 6B, suggesting coalescence of EdU-labeled viral DNA in the absence of pre-RCs.

Overall, simultaneous analysis of condensation of replicated viral DNA and development of LRCs suggests that viral DNA replication occurs in different areas of the nucleus, outside of LRCs. Replicated DNA condenses within LRCs as infection progresses on the platform of pre-RCs formed during the E phase of infection in the absence of viral replication, and gradual maturation of RCs may occur through the growth, movement, and coalescence of DNA-containing sites, as described in time-lapse studies of ICP8-GFP during the establishment of pre-RCs in HSV-1-infected cells [61]. These data also suggest that the maturation of RCs is not synchronous within the cell population and that the overall process appears to be rather inefficient. A significant proportion of infected cells failed to replicate DNA and express late proteins, and of the cells that did replicate DNA, only half were able to successfully form large RCs. Considering that pM25 is an important component of the tegument of the virion that accumulates in cytoplasmic organelles, it appears that the assembly of all the steps required for efficient release of the virion in Balb/ 3T3 cells is rather inefficient and that a relatively small number of cells are able to establish a complete manufacturing chain for productive release of virions.

### 3.6. Membraneless Assemblies Correlate with LLPS Propensity of MCMV Proteins

**Figure 7:**
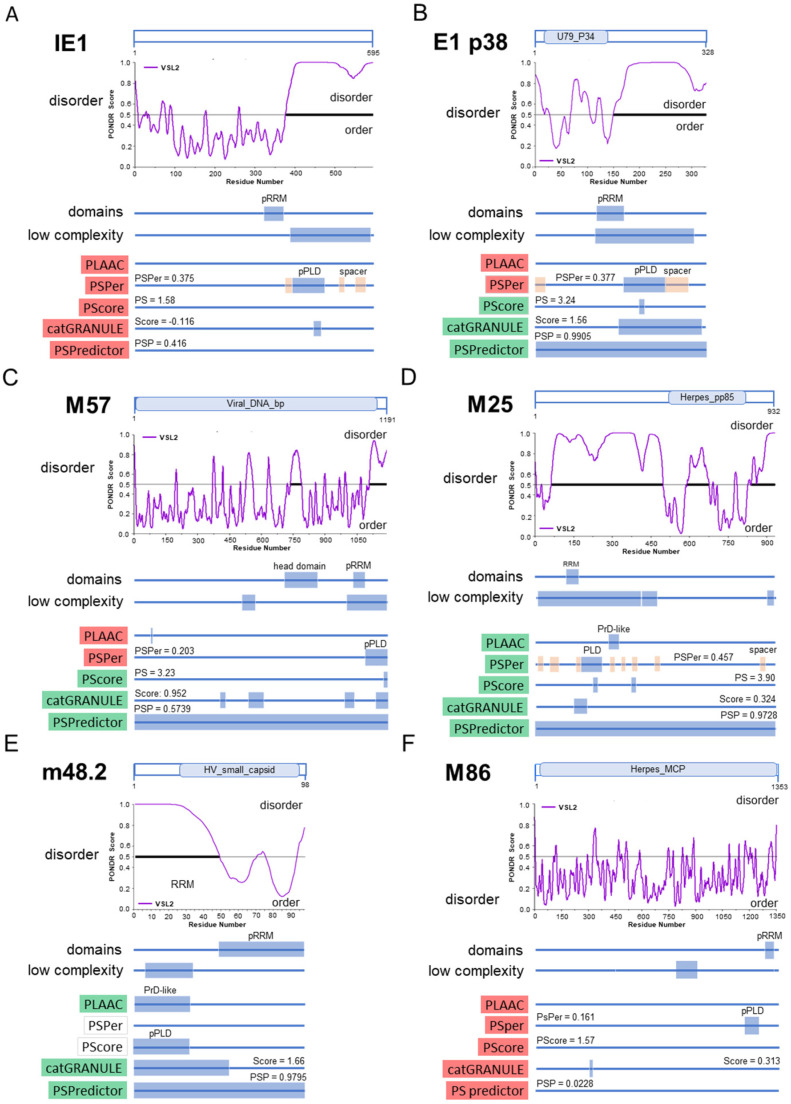
LLPS driver recognition and LLPS-specific prediction in MCMV proteins. pIE1 (**A**), p38 of E1 (**B**), pM57 (**C**), pM25 (**D**), pm48.2 (**E**), and pM86 (**F**) were analyzed for Pfam domains (oval boxes in the protein schemas), ordered and disordered regions (represented by the original PONDR VSL plot), LLPS-driving domains (blue boxes in the schema sequence of the protein identified by PSPer and SEG), and overall LLPS propensity using five LLPS prediction methods (shown below). In the schematics of the five LLPS prediction methods, the background color indicates the outcome of the predictions (left), with the green background representing a positive result and the red background representing a negative result, based on either the binary result (PLAAC) or the total score (four other methods). The white background color of PSPer and PScore for the m48.2 protein indicates that the web server tool requires a larger sequence to process. The blue boxes in the protein se-quence diagram denote the regions detected by the prediction methods. PLAAC detected PrD-like domains, PSPer PLD domains and spacer regions, PScore LLPS propensity regions, catGRANULE, and PSPredictor total propensity for LLPS (see Materials and Methods for more details). The box covering the entire protein sequence in PSPredictor shows the overall positive score, as this method does not assign regions. pRRM, putative RNA recognition motif.

Four of the five proteins used in this study (pE1, pM57, pM25, S-mCherry-SCP) showed MLAs, whereas pIE1 was mainly scattered in the nucleus. The appearance of these assemblies suggested that they might represent biomolecular condensates. Therefore, we next used bioinformatics tools to analyze the properties of these MCMV proteins that might be related to phase separation propensity. We focused on tools that can identify the protein architecture that can promote LLPS based on the primary sequence. We used PONDR [41] to identify IDRs, SEG [42] to predict LCRs, PLAAC [43] to identify prion-like LCRs, PSPer [44] to identify sticker and spacer regions and RNA recognition motifs (RRMs), and CIDER [62] to identify adhesion elements (distribution of hydrophobic residues). We also combined five prediction methods as proposed by Pancsa et al., 2021 [31] for the identification of LLPS drivers: PLAAC and PSPer, which evaluate the regions responsible for LLPS, PScore [45], which evaluates the propensity for LLPS at pi-pi contacts, catGRANULE algorithm [46,47] and the machine learning-based model PSPredictor [48], which predicts the overall propensity of a protein phase separation. The sequences of IE1, M112-113, M57, M25, and m48.2 were taken from UniProt, and the results describing the LLPS propensity of these proteins were summarized graphically in Figure 7.

Although the last third of pIE1 was identified as a highly disordered region of low complexity adjacent to a putative RNA recognition motif (pRRM) with a large putative prion-like domain (PLD) (Figure 7A), none of the five methods used to predict LLPS propensity (Figure 7A, bottom, highlighted in red) identified pIE1 as a protein with high propensity to LLPS. This is consistent with the diffuse nuclear distribution of pIE1 during all phases of MCMV infection.

The E1 (M112-113) gene of MCMV contains three exons that can give rise to at least four proteins (33, 36, 38, and 87 kDa) by alternative splicing, like its homolog UL112-113 in HCMV [19]. Half of the E1 p38 protein has a highly disordered LCR containing pRRM (Figure 7B). Three LLPS propensity prediction methods (Figure 7B, highlighted in green) identified E1 p38 as a protein with high LLPS propensity, including the machine learning program PSPredictor, which yielded a very high LLPS propensity score. Similar properties and scores were found for two other smaller (E1 p33 and p36) and large (E1 p87) forms (Figure S6). E1 p87 is mainly extended by an IDR. Thus, all forms of E1 protein have a high LLPS propensity, consistent with their condensation in the nucleus (Figure 1).

In contrast to pIE1 and pE1, pM57 did not have many regions of low complexity (Figure 7C). A large, disordered region of ~75 AA was located at the c-terminus and was upstream of the pRRM. PLAAC did not identify significant prion-like LCRs, whereas PSPer identified putative PLDs at the c-terminus. Neither PLAAc nor PSPer identified pM57 as an LLPS-promoting protein (Figure 7C, bottom, highlighted in red). However, PScore, cat-GRANULE, and PSPredictor analyses yielded relatively high scores (Figure 7C, bottom, highlighted in green) and classified pM57 as a protein that promotes phase separation. This is consistent with the intranuclear appearance of pM57 as circular assemblies at the end of the E phase and in the L phase within nuclear RCs (Figure 2).

The pM25 sequence exhibited a high degree of disorder, particularly in the 70–500 AA region with many LCRs, the pRRM region, prion-like domains, multiple spacer regions, and many sticker regions (Figure 7D). Based on the extent of disorder, it can be classified as an unfolded protein. PLAAC identified the PrD-like domain, PScore, catGRANULE, and PSPredictor gave values characteristic of a protein with a high propensity for LLPS, and the value determined by PSPer was close to the defined limit (Figure 7D). Therefore, it is very likely that pM25 undergoes phase separation, consistent with the formation of spherical assemblies in the E and L phases of infection in the nucleus, but also in the inner area of the cytoplasmic AC (Figure 3).

The first 50 AA region of the small capsid protein (SCP, pm48.2) was LCR and a highly disordered region with a PrD-like structure identified by PLAAC (Figure 7E). cat-GRANULE and PSPredictor yielded a high score, indicating that pm48.2 has a high propensity for phase separation. PSPer and PScore analysis could not be performed because at least 140 AA was required for analysis. Since in the infection experiments, we used recombinant MCMV expressing a construct containing the first 34 AA of SCP, the hemagglutinin tag (HA), mCherry, and the complete SCP sequence [20], we also analyzed this construct (Figure S7). This construct contains the duplicated LCR of SCP and showed a high propensity for LLPS after PSPredictor analysis, as expected. However, it did not yield a high PScore value. Nevertheless, pm48.2 has a sequence composition that classifies it as an LLPS driver, consistent with the formation of large S-mCherry-SCP assemblies in the L phase of infection. Since it is likely that pm48.2 (SCP) associates with pM86 (major capsid protein) in an equimolar ratio, as has been shown for their HCMV counterparts [63], we performed the same analysis for M86 (Figure 7F). This analysis showed that the five programs used to identify LLPS drivers yield very little disorder and LCRs, and very low scores. Therefore, it is likely that pm48.2 serves as a scaffold for LLPS segregation of the two proteins in the nuclear assemblies.

Overall, four proteins that form nuclear assemblies during the MCMV replication cycle (pE1, pM57, pM25, and pm48.2) demonstrated sequence features of LLPS drivers, suggesting that LLPS may be a mechanism for compartmentalization of nuclear replication events during the MCMV replication cycle.

### 3.7. Investigation of the Physical Properties of Membraneless Nuclear Compartments MCMV-infected Cells with 1,6-Hexanediol

1,6-Hexanediol (1,6-HD) is commonly used to determine the material properties of membranelles compartments in vivo [64,65]. We found that a 5-min exposure to 5% 1,6-HD was sufficient to dissolve most nucleolin condensates in uninfected Balb/3T3 cells without affecting cell viability, membrane blebbing, and cell shrinkage. Therefore, we applied this protocol to infected cells to test the physical properties of the observed nuclear condensates loaded with MCMV proteins.

At 6 and 16 hpi, treatment with 5% 1,6-HD for 5 min did not significantly alter the morphology of infected cells. However, at 24 hpi and 48 hpi, most infected cells detached after treatment with 1,6-HD. Reducing the concentration did not improve the attachment ability of the infected cells. Therefore, we examined the physical properties of MCMV protein-loaded condensates at 6 and 16 hpi, restricting our analysis to pE1- and pM25-loaded assemblies.

**Figure 8:**
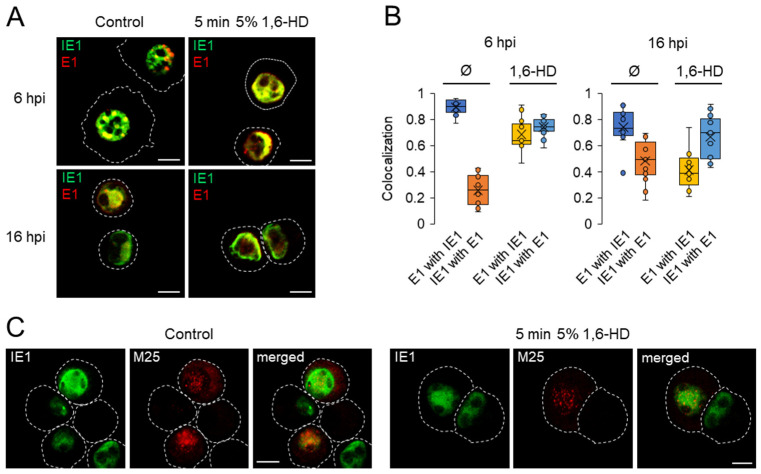
The effect of 1,6-hexanediol (1,6- HD) on the physical properties of pE1s and pM25 condensates formed in the early phase of infection. (**A**) Infected cells were treated 6 and 16 hpi with 5% 1,6-hexanediol (HD) for 5 min and stained for the distribution of pIE1 (green fluorescence) and pE1s (red fluorescence). Shown are the representative overlaid images. (**B**) Quantification of colocalization of pIE1 and pE1 in untreated and 1,6-HD-treated cells. 3D colocalization is based on the M1/M2 coefficients of pixel overlap measured over the Costes-algorithm thresholded z-stacks of confocal images. (**C**) Immunofluorescence visualization of pIE1 and pM25 in control and 1,6-HD-treated cells. Cell borders are indicated by fine dashed lines. Bars, 10 μm.

At 6 hpi, treatment with 1,6-HD dissolved the early stages of pE1s assemblies (patterns p1 and p2, Figure 1), and pE1s showed diffuse nuclear staining (Figure 8A). In control cells, pE1s was highly colocalized with pIE1, whereas only a fraction (~25%) of pIE1 colocalized with pE1s (Figure 8B), reflecting its diffuse nuclear distribution. After treatment with 1,6- HD, the colocalization of pIE1 with pE1s increased to ~70%, confirming the solubilization of pE1s assemblies and indicating liquid-like properties of pE1s assemblies.

By 16 hpi, pE1s assemblies were present in all forms (patterns p1–p4, Figure 1) in control cells. Patterns p3 and p4 occupied a large volume of the nucleus (Figure 8A), resulting in an increase in colocalization of pIE1 with pE1s to ~50% (Figure 8B). The pE1s condensates in the p1 and p2 patterns dissolved after 1,6- HD as described above, whereas the p3 and p4 patterns dissolved partially, resulting in a decrease in colocalization of pE1s with pIE1 and an increase in colocalization of pIE1 with pE1s (Figure 8B). These data suggest that the maturation of pE1s condensates leading to the development of RCs involves other interactions that alter the physical properties of the pE1-containing condensates.

In contrast to pE1, the pM25-containing nuclear condensates at 16 hpi were not dissolved by 1,6-HD (Figure 8C). The pM25 condensates at 24 hpi and especially at 48 hpi were much larger and better suited for 1,6-HD solubility analysis. These pM25 condensates were also 1,6-HD resistant, suggesting that pM25 condensates have more solid-like properties.

Overall, these data suggest that nuclear condensates containing MCMV proteins are organized by LLPS and that they are heterogeneous with respect to their physical properties. The condensates containing pE1s are liquid-like in the early stages, as indicated by solubility with 1,6-HD, and the later stages exhibit a more complex composition and physical state.

## 4. Discussion

This study provides a spatiotemporal analysis of nuclear events during the life cycle of MCMV by visualizing replicated viral DNA and five MCMV proteins. Nuclear events have been sporadically analyzed in MCMV-infected cells and addressed in several studies [16,17,18,19,20,21], but most understanding of events during MCMV infection relies on mimicking other beta-herpesviruses or even alpha-herpesviruses. Because the available data suggest that herpesviruses share the general principles of nuclear reorganization, studies of MCMV-infected cells may contribute to the overall understanding of the complex events associated with the establishment of the virus production chain in the nucleus. The same is true for the events in the cytoplasm.

The present study shows that the nucleus of MCMV-infected cells is compartmentalized by MLAs. This compartmentalization begins very early after infection and undergoes a maturation sequence as infection progresses until the complete nucleocapsid production chain is established. This chain includes at least compartmentalization of E- and L-gene transcription, viral DNA replication, and nucleocapsid assembly. Similar MLAs are often referred to as biomolecular condensates and can be formed by passive low-energy thermodynamic mechanisms and by active cellular processes [45]. Passive mechanisms can be based on many parameters, with LLPS at one end of the spectrum and the binding of molecules to static cellular structures (i.e., genomic DNA or RNA MLAs) at the other end [45]. Therefore, biomolecular condensation can be considered to be a form of nuclear compartmentalization during MCMV infection, as has been described for several RNA and DNA viruses [12,66], including herpesviruses [7,53,67,68,69,70,71,72]. Biomolecular condensation during MCMV infection can be achieved by a spectrum of mechanisms and purposes (see [10] for a detailed review), and our data support the concept [53] that nuclear compartmentalization can be achieved, at least in part, by LLPS-based biomolecular condensation.

In the absence of consensus for identifying LLPS propensity, we combined methods based on statistical analysis of primary protein sequence composition and computational machine learning analysis as recently proposed by Pancsa et al. [31]. We combined five methods developed for the detection of LLPS drivers that have been shown to be complementary in the detection of true LLPS [31]. However, these methods poorly detect LLPS driven by phosphorylation, other posttranslational modifications, or SLiM-domain interactions. LLPSs that require more than one protein for condensate formation, especially SLiM-mediated interactions, cannot be detected by currently available prediction methods.

**Figure 9:**
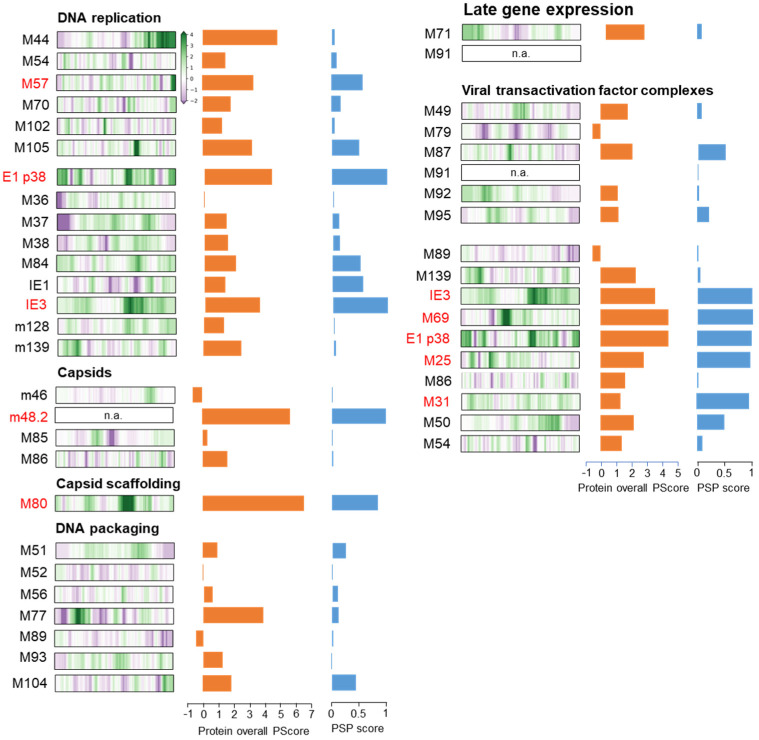
LLPS propensities of MCMV-encoded proteins contributing to nuclear events identified by the PScore and PSPredictor (PSP score) bioinformatics tools. Shown are the LLPS-propensity regions generated by PScore analysis and the total PScore and PSP score for each protein. Proteins identified as LLPS-prone proteins are highlighted in red.

The four proteins visualized by antibody tools in this study showed a high propensity for LLPS. According to the scaffolds and clients model [73], these proteins could be scaffolds that drive phase separation and form condensates, and clients may partition in these condensates. To explore this hypothesis, we extended our analysis of LLPS propensity to other MCMV proteins that contribute to nuclear events, using the same bioinformatics tools. The simplified result of this analysis is shown in Figure 9, using the results of two prediction methods, PScore and PSPredictor. This analysis shows that most viral proteins involved in nuclear processes have client properties and that LLPS-based nuclear compartmentalization can be driven by a relatively small number of scaffold proteins (Figure 9, highlighted in red).

Treatment with 1,6-HD showed that pE1 condensates, representing pre-RCs, exhibited a liquid-like state and increased in complexity at later stages of infection. In contrast, pM25 condensates formed in the early stages of infection did not dissolve by treatment with 1,6-HD, indicating that they are more solid. Unfortunately, we were not able to closely examine the physical nature of nuclear condensates in the infected cells in the late stages, after viral DNA replication and expression of viral structural proteins that load nuclear RCs. The nature of nuclear condensates containing pM57 and SCP cannot be properly assessed in cells that have resisted detachment after treatment with 1,6- HD. Considering that treatment with 1,6- HD reveals mainly hydrophobic interactions [64,65], which are only part of the spectrum driving LLPS [9,10,11], it is reasonable to expect that other mechanisms of biomolecular condensation are involved in the organization of such a large area of the cell representing RCs and compartmentalization within RCs. Therefore, further studies and identification of other tools are needed for more detailed characterization of all subsets of condensates within the nucleus, including conditions for monitoring solubility and liquid-like character and the transition from liquid-like to sloid-like states.

### 4.1. Nuclear Compartmentalization by MLAs

The earliest events of nuclear compartmentalization are associated with pE1. The E1 unit is the first of the expressed E genes [22,27,28], pE1 condenses in the nucleus and forms MLAs as early as 3–4 hpi. These pE1-laden MLAs, often referred to as pre-RCs, undergo a maturation sequence during the E phase of infection that ends with the formation of RCs after viral DNA replication (Figure 1C). A similar maturation sequence was recently described for HCMV E1 proteins [53], and likely as HCMV pE1, MCMV counterparts may induce LLPS around viral DNA prior to DNA replication. The pE1-loaded regions also accumulate IE1 (Figure 1) and IE3 [18] proteins, which may also contribute to LLPS (Figure 9), although a contribution from pIE1 is less likely since pIE1 also persists throughout the nucleoplasm. As E phase progresses, the MLAs loaded with pE1 grow and appear to fuse into larger structures, with pE1 eventually translocating to the periphery, resulting in the 1–3 large hollow structures that occupy nearly half of the nuclear space, similar to HCMV pE1 [53]. The hollow region is loaded with EdU-labeled replicated viral DNA (Figure 6H), but EdU-labeled viral DNA can also be found in cells with earlier stages of maturation of the pE1 structure, suggesting that viral DNA is replicated outside of pE1-laden MLAs. Previous studies have shown that the formation of MLAs and such a large structure is an intrinsic property of murine [18] and human [55] E1 proteins and can be formed in the absence of other viral proteins. Thus, E1 proteins can organize the earliest nuclear subcompartments by exploiting the propensity for LLPS and biomolecular condensation and retain this important function later during infection after viral DNA replication. Other early proteins, pM57 and pM25, are expressed later than E1 as the second set of E genes [22,28]. They also show a high propensity for LLPS and properties of condensation in the nucleus. pM25 accumulates within pE1-loaded structures prior to the establishment of RC [17] and condenses to pE1-free MLAs within the nucleus [16,17], including the hollow region of RCs. pM57 initially diffuses through the nucleus and condenses at the end of E phase throughout the pE1 cavity but not in the peripheral pE1-loaded regions of RCs, such as ICP8 [61] and UL57 [56], its counterparts in HSV-1 and HCMV-infected cells. Since pM57 has not been studied in MCMV-infected cells, it likely contributes to DNA replication and viral gene expression, as has been reported for ICP8 [70].

EdU-labeled replicated viral DNA also exhibited various forms of MLAs, including the final stage of condensation within RCs. pM57 is an obvious candidate for DNA condensation, as it is an essential component of the DNA replication machinery [74] and the only one that has LLPS propensity (Figure 9). However, the DNA replication site can also be formed by pIE3 and pE1, which are highly prone to LLPS (Figure 7 and Figure 9) and can form a replication initiation complex, as shown for HCMV pE1, which assembles pIE2 (homolog of MCMV IE3), pUL44, pUL54, and pUL84 [52]. Considering that pIE1 [21], pIE3 [18,21], and pM44 [18,19] associated with pE1 at nuclear localization sites, it can be assumed that DNA replication sites are organized by condensation initiated by pE1 and pIE3. Components of the DNA replication machinery accumulate in the pE1 domains, as shown for MCMV [18,19] and HCMV [55,56,57]. However, analysis of EdU incorporation patterns suggests that DNA replication may occur outside the E1 domain (Figure 6H).

At the later stage of RCs maturation, when pE1 is displaced to the periphery, most of the EdU-labeled DNA is located outside the pE1 domain, in the hollow region together with the MLAs of pM57. These regions are likely sites of viral genome transcription and L-gene expression, consistent with observations that DNA transcription occurs in separate regions within the nucleus in HCMV [56] and HSV-1 [75] infected cells. pIE1, pIE3, and pE1 may associate with pM79 and pM92 [76], two proteins that contribute to the organization of viral transactivator factor (vTF) complexes [49] required for L-gene expression [77] and may organize DNA transcription sites within the nucleus. Thus, pE1 might contribute to the organization of transcription centers at earlier stages of infection but not at later stages. Similarly, pM57 and pM25 could contribute to their organization, because both proteins are localized in condensates containing replicated viral DNA. It has been shown that pM25, but not pM57, binds to components of MCMV vTFs that are required for L gene expression [76]. Other proteins that bind to vTFs components, such as pM69 and pM31 [76], also exhibit high LLPS propensity (Figure 9) and may contribute to transcription center organization. Thus, the MLAs displayed by pM25 and pM57 in the nucleus may represent the sites of compartmentalization of the transcriptionally active MCMV genome in the L phase of infection.

HCMV and HSV studies have shown that the MLAs for viral DNA replication are not identical to the site of capsid assembly [6]. Candidate organizers of MLAs for capsid assembly include SCP (m48.2) and pM80 (capsid scaffolding protein), both of which have a high LLPS propensity (Figure 9). In cells infected with recombinant MCMV expressing S-mCherry-SCP, a high level of SCP condensation is found in peripheral regions of the nucleus, at sites distinct from MLAs with replicated viral DNA. Therefore, it is likely that due to high production, the surplus of S-mCherry SCPs is compartmentalized into storage sites that can be used to release SCPs for nucleocapsid formation. The HSV-1 capsid-forming protein VP26 also forms small foci at the periphery of RCs outside the areas where replicated viral DNA accumulates, which expand and coalesce over time at the periphery of the nuclei [58], suggesting similar behavior of capsid-forming proteins. Although SCPs from herpesviruses share common features, they are highly variable and likely have different functions [78]. Most SCPs from other herpesviruses do not exhibit features of LLPS propensity.

Sequestration of viral DNA likely occurs at a stage of nuclear maturation, as distinct localization of pM25 and EdU-labeled replicated DNA is observed in DNA clusters before the stage of large nuclear RCs (Figure 6G). After the formation of large RCs, most of the replicated viral DNA is located outside the pM25 MLAs. In these domains, pM25 might have a specific function in sequestering important cellular processes in MLAs, such as sequestering components of the cellular DNA replication machinery (PCNA) and p53 [17], since the absence of M25 has no effect on viral gene expression and DNA replication [16]. The information provided by M25 homologs of other herpesviruses to interpret the function of M25 in MCMV-infected cells is rather limited. Homologs of UL25 are essential for alpha-herpesvirus replication. pUL25 is involved in several steps during nuclear maturation of the capsid, including packaging of the viral genome and exit of the capsid from the nucleus [79]. Although pM25 and pUL25 are tegument proteins, MCMV M25 ORF is twice the size of HCMV UL25 ORF [80]. pUL25 is a true late protein like pM25l and mainly deposits in the cytosol. Thus, pM25l is organized into cytoplasmic MLAs in addition to nuclear MLAs, and the main function of pM25l may be to organize MLAs that sequester other tegument proteins in the cytoplasm AC. These cytoplasmic MLAs containing pM25l, are present only in the L phase of infection and in a limited number of cells.

The E1-hollow RCs appear to be the final stage of RC maturation and thus can be used as recognition marker to identify RCs. However, the large E1-hollow RCs are not formed in all cells. Thus, if terminal development of RCs is required for productive capsid maturation, many cells appear unable to form MLAs for productive capsid maturation.

### 4.2. Inefficient Compartmentalization of Nuclear and Cytoplasmic Virus Manufacturing

Our analysis of nuclear (this study) and cytoplasmic events [14,24,25,29] during MCMV infection showed that the establishment of nuclear and cytoplasmic virus production processes is not complete in all cells. This feature leads to difficulties in the systematic interpretation of imaging and biochemical data, especially after specific treatment of cellular processes. The nuclear factory consists of multiple operational centers organized as MLAs that assemble sequentially in the nucleus during infection to form a large RC. In addition, cytoplasmic production centers include both the formation of MLAs and the restructuring of membrane-bound organelles into the envelopment factory and export centers. An example of MLA is the cytoplasmic accumulation of pM25l, the most abundant tegument protein, as also described in other studies [16,17,59]. Both the nuclear and cytoplasmic centers are required for efficient virion assembly and egress and should be established in the virion-producing cell. This sequence cannot be achieved in every infected cell. Instead, it appears that only a relatively small proportion of cells can establish a fully functional production chain.

The IE and earliest E processes appear to be efficient, as shown by the expression of IE1 and E1 proteins in approximately 95% of cells after infection with 1 PFU per cell (MOI 10–20). The next step, expression of the second set of E genes (late E), is less efficient: i.e., pm06, pM57, and pM25 were expressed in 80–85% of infected cells. Replicated DNA can only be detected in 50–60% of infected cells, indicating that almost every other cell is unable to establish the machinery for replication of viral DNA. Accordingly, expression of true L genes essential for nucleocapsid and virion assembly, such as the pM55 and SCP proteins, can be detected in 50–60% of infected cells. Of the cells that replicated viral DNA, only half formed large replication centers. Even more, many cells that form the nuclear MLAs required for virus production fail to create the cytoplasmic environment required for the secondary envelopment. An example of this is the development of cytoplasmic pM25l MLAs, which have been detected in 25–30% of infected cells. It is known that M25 is not essential for virion release, but virions produced without M25 are smaller and less infectious [16]. Therefore, spatiotemporal coordination of viral gene expression and cellular processes is required to fully establish virus production. Our analysis of only a few components suggests that this process is highly inefficient and that only a small fraction of infected cells are able to release infectious virions. This is consistent with the discrepancies between the number of infected cells, the number of virions in the infected cell, and the number of virions released outside the infected cells observed over many years in HCMV and MCMV infection studies. The study with fluorescently labeled virions has also shown that the release of virions is a rather rare event [81].

Overall, this analysis suggests that the economics of viral infection should be considered. It appears that the infected cell produces many viral proteins in excess, leading to the development of storage or sequestration centers. These centers can only be organized by biomolecular condensation and release the sequestered components at the right time and in the right amount required to complete a particular step of viral production. However, it appears that not all processes are complete in all cells and only a limited number of cells are able to establish a complete production chain. Very little is known about this and should be taken into account.

## 5. Conclusions

Because MCMV infection is an important model for studying the life cycle of beta-herpesviruses, it is important to make further progress in analyzing the nuclear events associated with infection. In this study, we visualized five viral proteins (pIE1, pE1, pM25, pm48.2, and pM57) and replicated viral DNA to reveal nuclear events during MCMV infection. As expected, these events are similar to those described for other beta and alpha herpesviruses and contribute to the overall picture of herpesvirus assembly. This study focuses on compartmentalization of nuclear events that begin very early in infection, sequentially build a complex architecture during the E phase of infection (0–16 hpi), commonly defined as pre-RCs, and continue in L phase after viral DNA replication at 15–16 hpi to build nucleocapsid manufacturing centers, commonly defined as RCs. Compartmentalization is achieved through the formation of MLAs. Bioinformatics tools showed that four of these five proteins have a high propensity for LLPS, suggesting that LLPS may be a mechanism for compartmentalization of the infected nucleus. One of these proteins (pM25), which is also expressed in a cytoplasmic form (pM25l), exhibited MLAs in the cytoplasm, suggesting that LLPS-induced compartmentalization may be used for organization of the cytoplasmic AC. Therefore, the formation of MLAs is a fundamental mechanism for compartmentalization of the nuclear and cytoplasmic virus production chain. Establishment of the chain requires high spatiotemporal coordination of the high supply of virus-encoded proteins and host cell functions.

Our analyses of the five proteins and viral DNA indicates that many cells do not complete maturation processes efficiently and a rather limited number of cells establish virus production and release. This finding should be considered when planning high-throughput and single-cell analyses. In addition, proper selection of a sufficient number of checkpoints and biomarkers for these processes can help in planning single-cell studies to clarify the arrangement of host cell factors and host cell functions required for efficient virus production. This is particularly true for cells that cannot establish a productive viral production chain and could provide important insights into understanding latency and reactivation, a common and intriguing feature of herpesviruses.

## Data Availability

The raw data supporting the conclusions of this article will be made available by the authors without undue reservation.

## Supplementary Materials

The following supporting information can be downloaded at: https://www.mdpi.com/article/10.3390/v15030766/s1, Figure S1: Expression pattern of pIE1; Figure S2: Western blot analysis of the expression of pIE1 and pE1s during the MCMV replication cycle; Figure S3: Original superimposed raw blots and unprocessed ECL images of M57 and β-actin used as a representative Western blot in Figure 2C of the manuscript; Figure S4: Original superimposed raw blots and unprocessed ECL images of M25 and β-actin used as a representative Western blot in Figure 3C of the manuscript; Figure S5: Expression kinetics of the S-mCherry-SCP protein in the course of MCMV infection; Figure S6: LLPS driver analysis of four forms of E1 proteins using bioinformatics tools; Figure S7: LLPS driver recognition and LLPS-specific prediction in the S-mCherry-SCP protein.
