# Supplementary material for: Membraneless Compartmentalization of Nuclear Assembly Sites during Murine Cytomegalovirus Infection
Source: Viruses. 2023 Mar 16;15(3):766. doi: 10.3390/v15030766 (PMC10053344; doi:10.3390/v15030766)
Supplement: Supplementary file 1 [file viruses-15-00766-s001.zip › viruses-2157929-supplementary.pdf]

Supplementary material

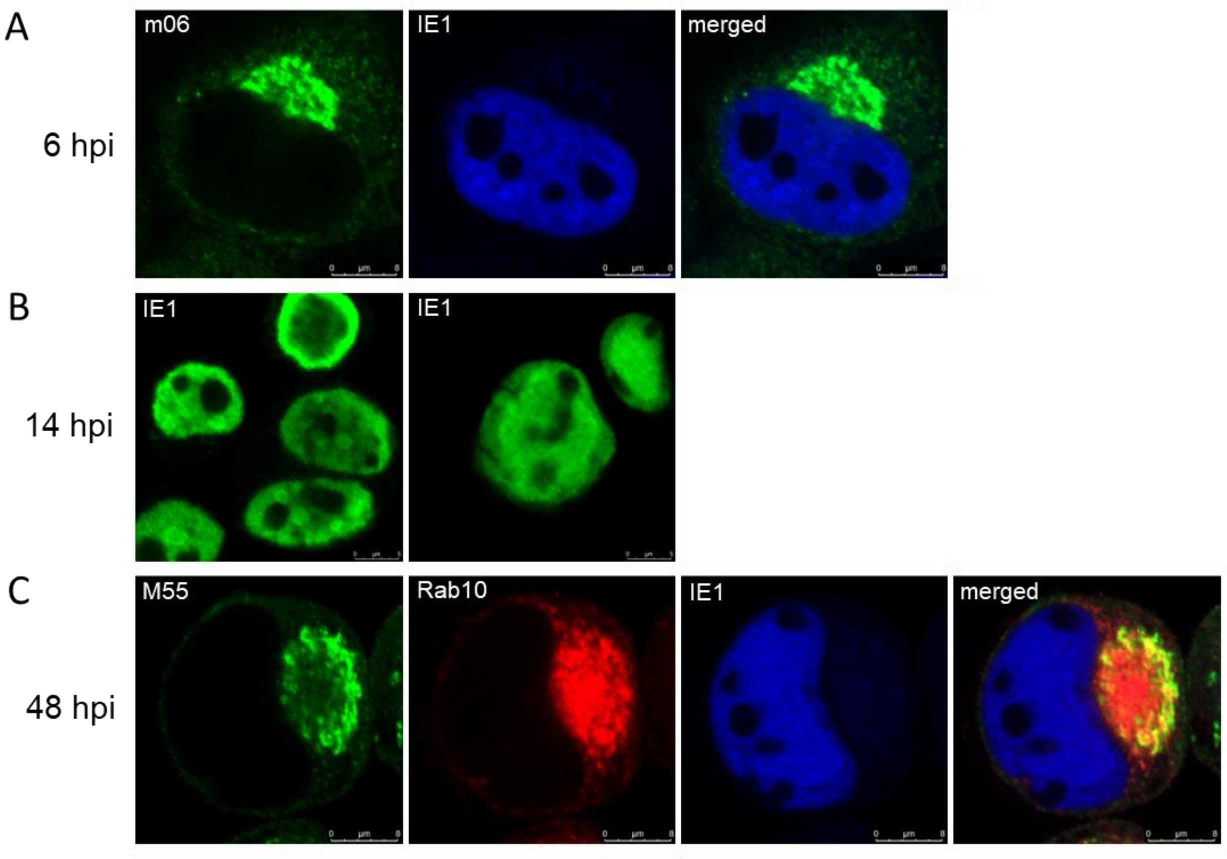

Figure S1. Expression pattern of pIE1. Balb3T3 cells were infected with Δm138-MCMV at a MOI of 10 and stained for IE1. (**A**) Visualization of nuclear expression of pIE1 and cytoplasmic expression of pm06 at 6 hpi. (**B**) Pattern of pIE1 staining at the end of E phase of infection (14 hpi). (**C**) Nuclear distribution of pIE1 and visualization of the cytoplasmic assembly compartment (AC) by staining for the virus-encoded glycoprotein M55 localized in the outer-AC and over-recruited host-cell protein Rab10 localized in the inner-AC.

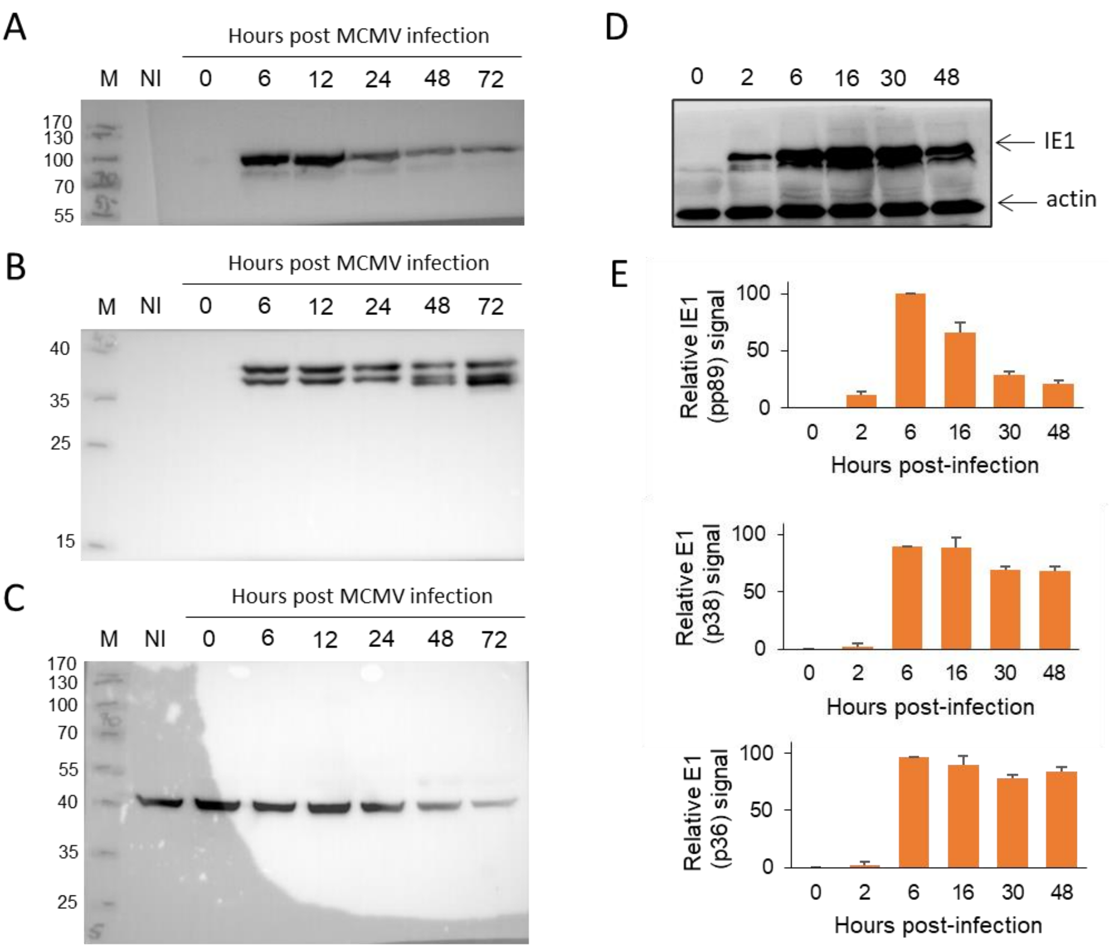

Figure S2. Western-blot analysis of the expression of pIE1 and pE1s during the MCMV replication cycle. (**A-C**) Superimposed original raw blots and unprocessed ECL images of pIE1 (**A**), pE1s (**B**), and β-actin (**C**) used as representative Western blots in Figure 1B of the manuscript. (**A-B**) The original raw blot was cut below the 55 kDa marker line, and the upper portion was used to stain IE1 and the lower portion to stain E1. The IE1 protein is shown in the 89 and 72 kDa forms, while the E1 proteins are shown in the 38, 36, and 34 kDa forms. The molecular weight markers used in these experiments are PageRuler Prestained Protein Ladder (Thermo Scientific, product: 26616, LOT:00515508). M, marker line; 0-72, preparation time of infected cell samples. (**D**) IE1 protein expression can be detected as early as 2 hours after infection. Image of a Western blot stained simultaneously for IE1 protein and β-actin. (**E**) Expression levels of pIE1 and pE1s during the MCMV replication cycle. Densitometric analysis of Western blots (5 independent experiments) was performed using Image J 1.53 software and normalized to the actin signal, which was used as a loading control. First, the normalization factor for each lane was calculated using the following formula: Lane normalization factor = observed actin signal for each lane/highest observed actin signal for the blot. Then, the normalized experimental signals were calculated as the ratio between the observed experimental signal and the lane normalization factor. Data represent means ± SEM for IE1 pp89 and E1 p38 and p36.

Balb3T3 cells were placed in 12-well plates ($7x10^4$ cells/well) and infected the next day with Δm138-MCMV (virus 95.15) at a MOI of 10. Samples were collected immediately (0) and at various time points post-infection. Cells were detached with a short trypsin/EDTA treatment (120 µl/well), trypsin was neutralized with 1.2 ml tissue culture medium containing 10% FCS, pelleted at 2,200 rpm for 2.5 min, and washed twice with PBS. The pellet was lysed on ice in 20 µL of lysis buffer (RIPA buffer; Pierce RIPA Buffer, Prod 89900, LOT PI208053, Thermo Scientific) containing PMSF and the cocktail of protease inhibitors and centrifuged at 13,000g for 15 min at 4°C. Supernatants were supplemented with 5 µL of 5X reducing SDS buffer and boiled at 95°C for 10 min. Samples were separated at 130 V on a 12% gel (Acrylamide Roth) using BIO RAD PowerPac Universal. The gels were transferred to a PVDF membrane (Merck Milipore, size: 0.45 µm, Immobilon-P Transfer Membranes CAT: IPVH00010, LOT: R6PA1239C) for 2 hr at 80 V. After blotting, membranes were cut below the 55kDa marker line, washed with 1xTBS and blocked with 0.5% Blocking Regent (Roche) for 60 minutes and the upper part was incubated with anti-IE1 monoclonal antibody (clone CROMA 101, 1mg/ml) and the lower part was incubated with mouse anti-E1 monoclonal antibody (clone CROMA103, 1mg/ml). The separate membrane was stained with anti-actin. The primary antibodies were resuspended in 0.5% blocking buffer and incubated overnight at 4°C. After washing three times for 15 minutes with TBST (TBS with 0.05% Tween), membranes were incubated for one hour at room temperature with the anti-mouse antibody POD (Jackson 1:50 000 diluted 1:1000 of 1:50), washed three times (10 minutes each time) with TBST, and incubated with the SignalFire (TM) Plus ECL reagent (Cell Signaling, 12630S, LOT no. 6) and treated with the SignalFire (TM) Elite ECL reagent (Cell Signaling, 12757P, lot #7).

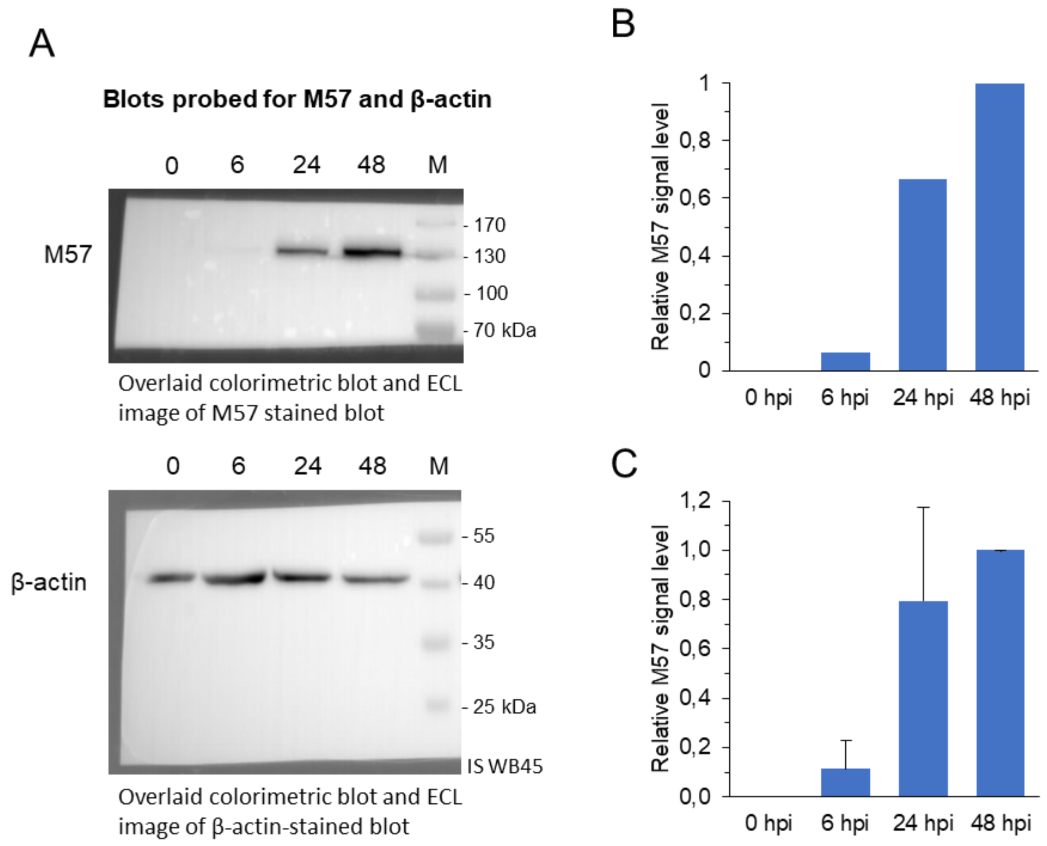

Figure S3. Original superimposed raw blots and unprocessed ECL images of M57 and β-actin used as a representative western blot in Figure 2C of the manuscript. (**A**) The original raw blot was cut below the 70 kDa marker line and used to stain M57 and IE1 β-actin. The M57 protein is shown in a 131 kDa form. The molecular weight markers used in these experiments are PageRuler Prestained Protein Ladder (Thermo Scientific, Product: 26616, LOT: 00515508). M, marker line; 0-48, preparation time of infected cell samples. (**B**) Denistometric analyis of western blot signals shown in A, analyzed by Image J 1.53 software and normalized to actin signal used as a loading control. We first calculated the normalization factor for every lane according to the formula: Lane normalization factor = Observed signal of actin for every lane/Highest observed signal of actin for the blot. Following that, Normalized experimental signals were calculated as the Observed experimental signal/Lane normalization factor ratio. (C) Average M57 levels determined on 5 independent experiments.

Balb3T3 cells were placed in 12-well plates (7×10^4 cells/well) and infected the next day with Δm138-MCMV (virus 95.15) at MOI of 10. Samples were collected immediately (0) and 6, 24 and 48 hours after infection. Cells were detached with a short trypsin/EDTA treatment (120 µl/well), trypsin was neutralized with 1.2 ml tissue culture medium containing 10% FCS, pelleted at 2,200 rpm for 2.5 min, and washed twice with PBS. The pellet was lysed on ice in 20 µL of lysis buffer (RIPA buffer; Pierce RIPA Buffer, Prod 89900, LOT PI208053, Thermo Scientific) containing PMSF and the cocktail of protease inhibitors and centrifuged at 13,000g for 15 min at 4°C. Supernatants were supplemented with 5 µL of 5X reducing SDS buffer and boiled at 95°C for 10 min. Samples were separated at 130 V on a 10% gel (Acrylamide Roth) using BIO RAD PowerPac Universal. The gels were transferred to a PVDF membrane (Merck Milipore, size: 0.45 µm, Immobilon-P Transfer Membranes CAT: IPVH00010, LOT: R6PA1239C) for 2 hours at 80 V. After blotting, membranes were cut below the 70 kDa marker line, washed with 1xTBS, blocked with 0.5% Blocking Regent (Roche) for 60 min, and the upper portion incubated with anti-M57 monoclonal antibody (clone M57.02, 1mg/ml, LOT:001; 1:500) and the lower portion incubated with mouse anti-actin. The primary antibodies were resuspended in 0.5% blocking buffer and incubated overnight at 4°C. After washing three times for 15 min with TBST (TBS containing 0.05% Tween), membranes were incubated for 1 hour at room temperature with anti-mouse POD (Jackson 1:50 000 diluted 1:1000 from 1:50 stock), washed three times (10 min each) with TBST and treated with SignalFire (TM) Plus ECL Reagent (Cell Signaling, 12630S, LOT No:6) and SignalFire (TM) Elite ECL Reagent (Cell Signaling, 12757P, Lot No:7).

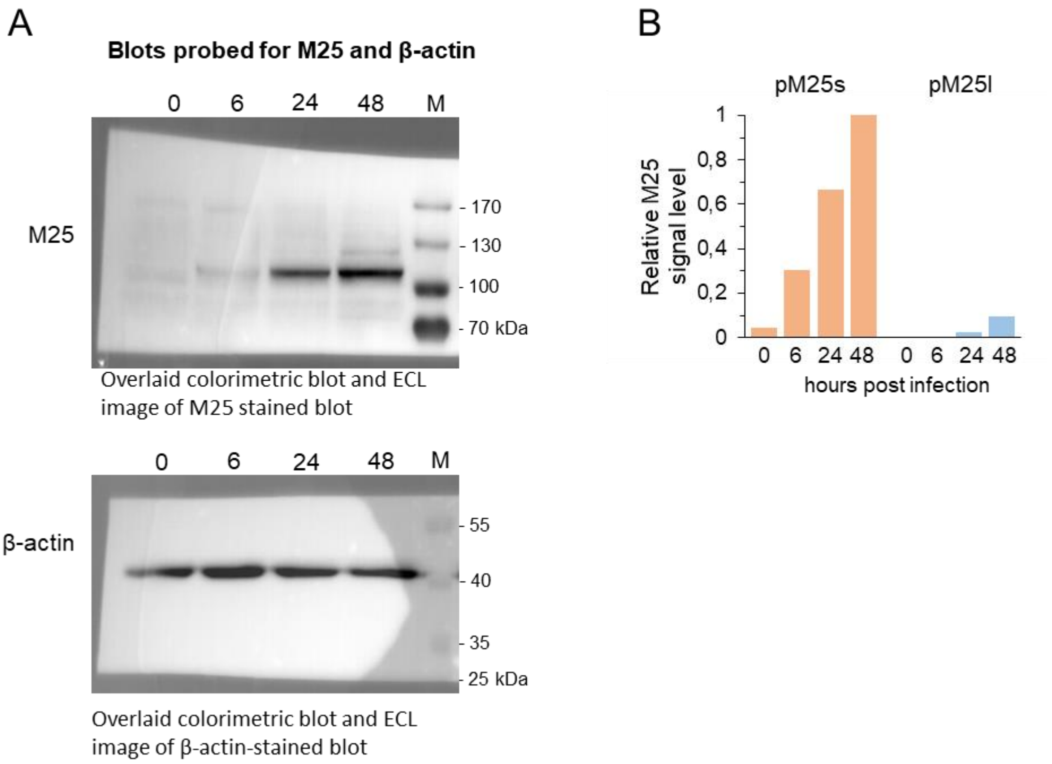

Figure S4. Original superimposed raw blots and unprocessed ECL images of M25 and β-actin used as a representative western blot in Figure 3C of the manuscript. (A) The original raw blot was cut below the 70 kDa marker line and used to stain M25 and IE1 β-actin. The M25 protein is shown as a tegument protein of 130 kDa (pM25l) and nonstructural isoform of 105 kDa (pM25s) (Kutle et al., 2020). The molecular weight markers used in these experiments are PageRuler Prestained Protein Ladder (Thermo Scientific, Product: 26616, LOT: 00515508). M, marker line; 0-48, preparation time of infected cell samples. (B) Densitometric analysis of western blot signals shown in A, analyzed by Image J 1.53 software and normalized to actin signal used as a loading control. We first calculated the normalization factor for every lane according to the formula: Lane normalization factor = Observed signal of actin for every lane/Highest observed signal of actin for the blot. Following that, Normalized experimental signals were calculated as the Observed experimental signal/Lane normalization factor ratio. (C) Average M57 levels determined on 5 independent experiments.

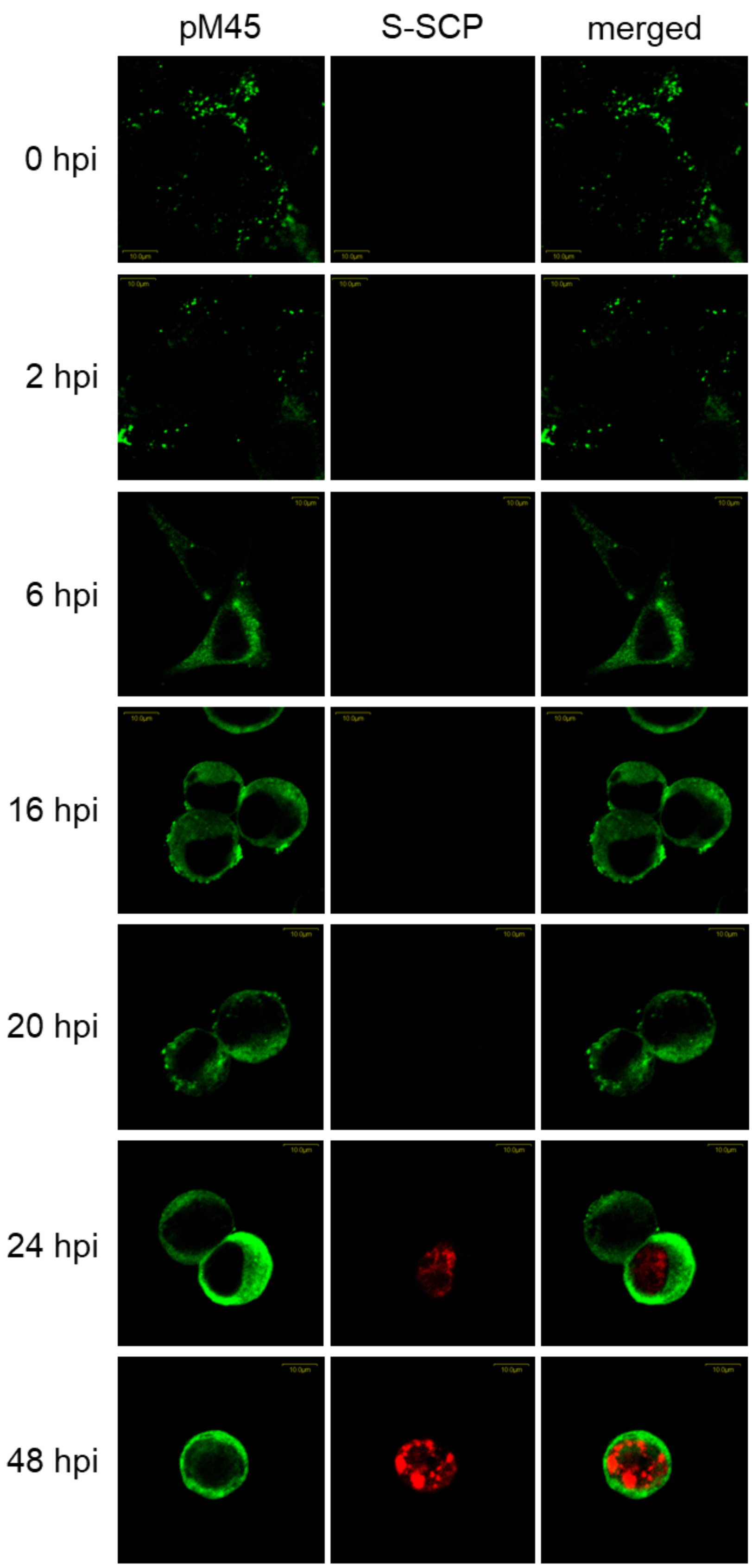

Figure S5. Expression kinetics of the S-mCherry-SCP protein in the course of MCMV infection. Balb 3T3 cells were infected at MOI of 10 with S-mCherry-SCP-MCMV, fixed at different time points, and stained for immunofluorescence analysis with mouse IgG_1-mAb against pM45, followed by staining with AF^{488}-conjugated anti-mouse IgG1 and confocal microscopy analysis. Shown are representative images through the focal plane. Bars, 10 μm.

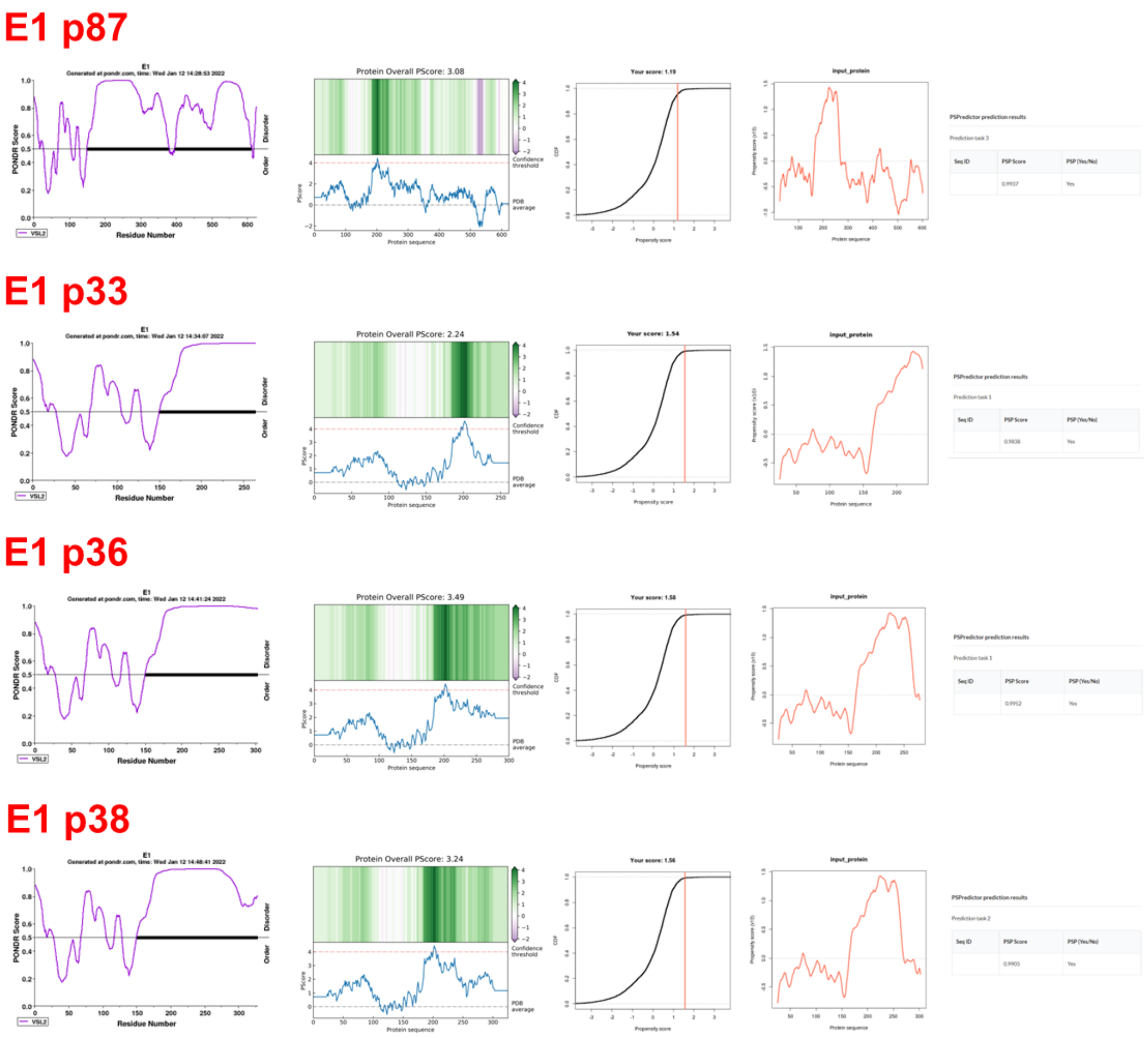

Figure S6. LLPS driver analysis of four forms of E1 proteins using bioinformatics tools. The sequences of E1 p33, p36, p38, and p87 were obtained from UniProt and analyzed by PONDR for identification of disordered domains, and by PScore, catGRANULE and PSPredictor tools for identification of LLPS propensity. Shown are original images returned after analysis by these tools.

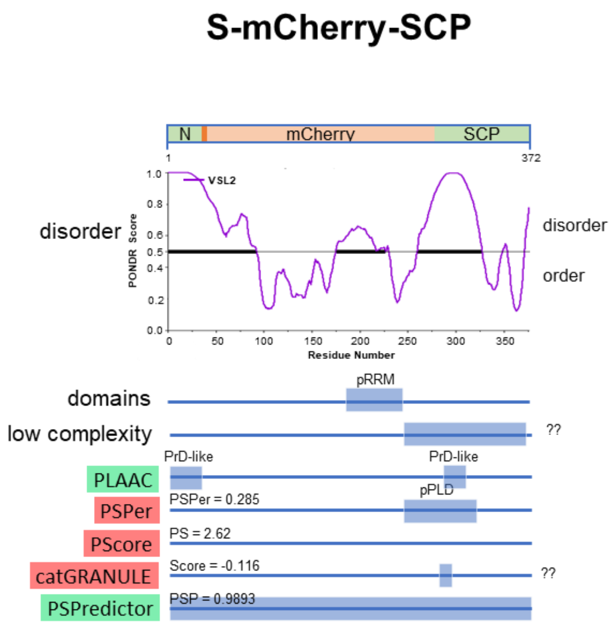

Figure S7. LLPS driver recognition and LLPS-specific prediction in the S-mCherry-SCP protein. The protein scheme gives the structure of the construct: N-terminal 34 amino acids (N) of SCP (encoded by m48.2 MCMV gene), hemagglutinin tag, complete sequence of mCherry protein and complete sequence of SCP [19]. Ordered and disordered regions are shown by the original plot derived from the PONDR VSLs. The blue boxes in the scheme of protein sequences represent LLPS driver regions. The LLPS driving domains and low complexity regions were identified by PSPer and SEG, respectively. pRRM, putative RNA recognition motif. Five LLPS prediction methods are shown below. The background color of the prediction method names, the regions detected by the prediction method in the protein sequence scheme, and the total score indicate the result of the predictions. The green background color represents a positive result, and the red background color represents a negative outcome of the predictions, based on either the binary result (PLAAC) or the total score (four other methods). PLAAC detected PrD-like domains, PSPer PLD domains and spacer regions, PScore LLPS-propensity areas, catGRANULE, and PSPredictor the overall propensity for LLPS. The boxes show the regions detected by the methods. The box covering the entire protein sequence in PSPredictor shows the overall positive score, as this method does not assign regions.
